# MasitinibL shows promise as a drug-like analog of masitinib that elicits comparable SARS-Cov-2 3CLpro inhibition with low kinase preference

**DOI:** 10.1038/s41598-023-33024-2

**Published:** 2023-04-28

**Authors:** Olanrewaju Ayodeji Durojaye, Nkwachukwu Oziamara Okoro, Arome Solomon Odiba, Bennett Chima Nwanguma

**Affiliations:** 1 MOE Key Laboratory of Membraneless Organelle and Cellular Dynamics, Hefei National Laboratory for Physical Sciences at the Microscale, University of Science and Technology of China, Hefei, 230027 Anhui China; 2 School of Life Sciences, University of Science and Technology of China, Hefei, 230027 Anhui China; 3 Department of Chemical Sciences, Coal City University, Emene, Enugu State Nigeria; 4 Department of Pharmaceutical and Medicinal Chemistry, Faculty of Pharmaceutical Sciences, University of Nigeria, Nsukka, 410001 Nigeria; 5 Department of Molecular Genetics and Biotechnology, University of Nigeria, Nsukka, 410001 Enugu State Nigeria; 6 Department of Biochemistry, Faculty of Biological Sciences, University of Nigeria, Nsukka, 410001 Enugu State Nigeria

**Keywords:** Computational biology and bioinformatics, Drug discovery, Drug screening, High-throughput screening, Virtual screening

## Abstract

SARS-CoV-2 infection has led to several million deaths worldwide and ravaged the economies of many countries. Hence, developing therapeutics against SARS-CoV-2 remains a core priority in the fight against COVID-19. Most of the drugs that have received emergency use authorization for treating SARS-CoV-2 infection exhibit a number of limitations, including side effects and questionable efficacy. This challenge is further compounded by reinfection after vaccination and the high likelihood of mutations, as well as the emergence of viral escape mutants that render SARS-CoV-2 spike glycoprotein-targeting vaccines ineffective. Employing de novo drug synthesis or repurposing to discover broad-spectrum antivirals that target highly conserved pathways within the viral machinery is a focus of current research. In a recent drug repurposing study, masitinib, a clinically safe drug against the human coronavirus OC43 (HCoV-OC43), was identified as an antiviral agent with effective inhibitory activity against the SARS-CoV-2 3CLpro. Masitinib is currently under clinical trial in combination with isoquercetin in hospitalized patients (NCT04622865). Nevertheless, masitinib has kinase-related side effects; hence, the development of masitinib analogs with lower anti–tyrosine kinase activity becomes necessary. In this study, in an attempt to address this limitation, we executed a comprehensive virtual workflow in silico to discover drug-like compounds matching selected pharmacophore features in the SARS-CoV-2 3CLpro-bound state of masitinib. We identified a novel lead compound, “masitinibL”, a drug-like analog of masitinib that demonstrated strong inhibitory properties against the SARS-CoV-2 3CLpro. In addition, masitinibL further displayed low selectivity for tyrosine kinases, which strongly suggests that masitinibL is a highly promising therapeutic that is preferable to masitinib.

## Introduction

SARS-CoV-2 (severe acute respiratory syndrome), the cause of COVID-19 (coronavirus disease 2019), has resulted in 627,104,342 confirmed cases and 6,567,552 deaths globally as of the end of October 2022, according to the World Health Organization (WHO)^1^. The urgency to mitigate this acute infection justifies the race to develop or repurpose therapeutics, especially with the continuing emergence of new variants with varying degrees of infectivity and disease severity. These efforts yielded several options with Emergency Use Authorization (EUA) status for treating COVID-19, of which some include dexamethasone^2^, RNA-dependent RNA polymerase inhibitors (molnupiravir and remdesivir), 3CLpro inhibitors (nirmatrelvir), monoclonal antibodies (MABs), and convalescent plasma^3^. However, most of these treatment options displayed major setbacks in large clinical trials in hospitalized patients, either exhibiting uncertain efficacy or serious side effects^4,5^. Moreover, the SARS-CoV-2 virus mutates frequently, and this is an obstacle to some potential drugs, and the emergence of viral escape mutants that render SARS-CoV-2 spike glycoprotein-targeting vaccines ineffective remains a possibility^6,7^. This challenge necessitates the search for broad-spectrum antivirals that will also be effective against possible future variants. Tackling this challenge will require targeting a highly conserved and stable region of the SARS-CoV-2 virus core protein machinery^8^. The main viral protease, 3CLpro (also known as Mpro or nonstructural protein 5 [nsp5]), is responsible for cleaving translated proteins into active proteins^9^. Hence, it is an attractive target for antiviral drugs because it is indispensable for viral replication and is well conserved among coronaviruses^10^. Furthermore, drugs that target 3CLpro are unlikely to be unaffected by possible spike protein mutations caused by immunological pressure following natural infection or vaccination. Hence, targeting 3CLpro by de novo drug design or drug-repurposing remains a viable therapeutic path to be explored^11,12^.

Global efforts were accelerated nine months after the COVID-19 pandemic started, which allowed us to reach the point where preclinical and early clinical evidence were available for the vaccines currently in use^13^. About 128 candidate vaccines are now undergoing clinical examination; 194 candidate vaccines are undergoing preclinical evaluation; and more than 12 vaccines have finished phase 3 clinical trials, gained phased-in WHO approval, or been granted emergency authorization as of October 22, 2021^13^. The basis of every authorized vaccination depends on the coronavirus spike protein (S) being expressed as the primary immunogenic antigen of the SARS-CoV-2 virus, inducing an immunological response that results in the production of certain antibodies that are capable of neutralizing the virus^14^. The vaccines being tested in clinical trials use both conventional approaches, such as recombinant antigen subunits with immunogenic viral epitopes (45 vaccine candidates), purified whole-inactivated viruses (17 vaccine candidates), live attenuated viruses (2 vaccine candidates), virus-like particle vaccines (5 candidate vaccines), and next-generation vaccination platforms, such as RNA- and DNA-based formulations (35 vaccine candidates), and the bacterial antigen-spore vaccine (25 vaccine candidates)^13^.

De novo drug synthesis is an arduous process; hence, drug repurposing screens have been employed by numerous studies to identify existing approved drugs that can be used for other purposes^15^. Recently, one of these drug repurposing screens identified masitinib (the top candidate out of 1900 candidates) to be very effective against SARS-CoV-2, completely inhibiting 3CLpro activity and blocking replication of SARS-CoV-2 in cells^16^. Masitinib, a clinically safe drug against OC43 (the cause of the common cold), is an orally bioavailable tyrosine kinase inhibitor^17^. Masitinib has been considered for a broad range of applications in human diseases, including the treatment of cancer^18^, Alzheimer’s disease^19^, asthma^20^, amyotrophic lateral sclerosis^21^, multiple sclerosis^16^, and mast cell tumors in dogs^22^. Evidence from biochemical and X-ray crystallography shows that masitinib acts as a competitive inhibitor of SARS-CoV-23CLpro^23^. Masitinib reduced lung inflammation and displayed a > 200-fold reduction in viral titers in the lungs and noses of mice infected with SARS-CoV-2^16^. Interestingly, masitinib also showed in vitro effectiveness against B.1.1.7, B.1.351, and P.1 variants of SARS-CoV-2. The results further showed that masitinib reduced SARS-CoV-2 viral load in mice (> 99% on day six) and reduced inflammatory signatures, demonstrating potential benefits for survival^24^. Masitinib has been registered with clinicaltrials.gov (identifier: NCT04622865) to test its combined efficacy with isoquercetin on hospitalized patients^23^. Nevertheless, masitinib has limitations, such as kinase-related side-effects, and the study by Drayman et al.^23^ recommended the development of masitinib analogs with lower anti–tyrosine kinase activity, which would be beneficial to reduce its reported side-effects. Hence, we sought potential candidates with more advantages that would overcome the limitations of masitinib and also elicit better virucidal activity than masitinib.

In this study, we employed a comprehensive virtual screening workflow protocol to screen several chemical compound databases for drug-like compounds matching selected pharmacophore features in the SARS-CoV-2 3CLpro-bound state of masitinib, from which significant hits were found. Similar pipelines have been employed in the recent design and synthesis of potent SARS-CoV-2 3CLpro inhibitors^25^. We identified a novel lead compound through protein–ligand interaction fingerprinting and a comprehensive molecular dynamics simulation with the 3CLpro-bound masitinib, which strongly suggested that the novel lead compound (a structural analog of masitinib, named masitinibL in this study) is a highly potent inhibitor of the SARS-CoV-2 3CLpro, which further displayed a relatively lower anti–tyrosine kinase activity.

## Materials and methods

### 3CLpro preparation and generation of receptor grid

The 3-dimensional crystal structure of the SARS-CoV-2 3CLpro in complex with masitinib was obtained from the RSCB Protein Data Bank^26^. Thereafter, to ensure structural correctness and high confidence, we employed the Schrödinger Maestro suite (version 11.8) for structural preparation^27^. Preprocessing was carried out through the assignment of bond orders and the addition of hydrogen. Options to create zero-order bonds to metals, create disulfide bonds, and convert selenomethionines to methionines were also selected, along with the filling of missing loops and sidechains using Prime. Furthermore, the temini were capped, water molecules beyond 5 angstroms were also deleted, and het states were generated at a pH of 7.0 ± 2.0 using Epik^28^. The energy minimization step was then carried out through the application of the OPLS3e force field^29^. Upon completion of the protein preparation protocol, we generated the receptor grid where the docked ligands will be confined during the virtual screening process using the receptor grid generation function of the Schrödinger Maestro suite. To generate an ideal grid, the bound masitinib was selected in the receptor tab, and other parameters were left at their default values. The generated X, Y, and Z coordinates for the docking grid are 13.66, 4.42, and 24.36, respectively.

### Pharmacophore generation and chemical compound database screening

Using the bound pose of the co-crystallized masitinib as a template, important pharmacophore features were identified and selected for screening through the ZINCPharmer platform^30^. The generated pharmacophore features were used to screen the chemical compound databases for compounds matching the properties of the selected features, taking into consideration Lipinski’s rule of five for drug likeness^31^. The chemical compound databases screened (based on pharmacophore-matching) include the CHEMBL30, ChemDiv, ChemSpace, Mcule, Mcule-ultimate, MolPort, NCI Open Chemical Repository, WuXi LabNetwork, and the ZINC databases. A maximum of one hit per molecule and one hit per conformation reduction parameter were applied in order to narrow down the number of hits while satisfying the minimum requirement for drug likeness. The parameters set include a maximum molecular weight of 500, a maximum number of rotatable bonds of 10, a maximum LogP value of 5, a maximum of 10 hydrogen bond acceptors, and a maximum of 5 hydrogen bond donors. An in-house Python script was employed to address redundancy across databases.

### Ligand preparation

Having obtained all pharmacophore-matching chemical compounds from their respective databases, we used the Schrödinger Maestro Suite LigPrep module for all ligand preparation steps^32^. Multiple conformations (at most 32) of each ligand at a pH of 7.0 ± 2.0 were generated using Epik^28^, while also generating tautomeric stereoisomers. The 3D structure of masitinib in SDF format was subjected to a similar protocol that returned three conformers. All ligands were desalted, and other parameters were retained in their default settings. The Qikprop program screening was deselected before running LigPrep since Lipinski-based ligand filtering had been included during the compound database screening process. The LigPrep output was appended to the workspace upon completion and saved in Maestro format.

### Virtual screening and MM-GBSA postprocessing

The ligand preparation process generated a huge number of compounds due to the permitted maximum limit of 32 conformers for each ligand. Hence, all the hits were taken through a cleanup protocol involving a 3-step virtual screening workflow followed by the MM-GBSA postprocessing to narrow down the number of hits. The 3-step virtual screening protocol includes the HTVS (High Throughput Virtual Screening)^33^, the SP (Standard Precision Module), and the XP (Extra Precision Module)^34^. For the HTVS step, we maintained the default setting of 10% retention of the top 10 compounds. These sets of compounds were passed on to the standard precision module, and the same default setting as used in the HTVS was maintained. Additionally, for both the HTVS and the SP steps, only one pose was allowed to be generated per compound after docking. The output of the SP step was then passed on to the XP module, where 5% was retained. The Molecular Mechanics Generalized Born Surface Area (MM-GBSA) protocol was conducted in an attempt to make a final selection after the ranking of the three generated pose outputs from the extra precision docking step. The binding affinity calculation based on pose ranking was conducted using the Schrödinger suite Prime MM-GBSA module^35^, which implements several methods such as the OPLS molecular mechanics energies, the presence of a non-polar solvent, and a SGB solvation model. The MM-GBSA was calculated using the equation:

$$ \Delta {\text{Gbind}} = {\text{Gcomplex}} - {\text{Gpotein}} + {\text{Gligand}} $$

$$ {\text{where}}\;{\text{G}} = {\text{EMM}} + {\text{VSGB}} + {\text{GNP}}{.} $$

### Identification of lead compound

In order to select the most ideal group of lead compounds for further analysis, we conducted a protein–ligand interaction fingerprinting (PLIF) study on the top 10 outputs from the MM-GBSA ranking using the Aupossom software^36^. Upon docking completion, the receptor and ligand files were converted to MOL2 format for Aupossom upload. The contact type for analysis was set to hydrogen bond (hb), while the module threshold for partial charges was set at 0.5. Other parameters were retained in their default settings to run the program. Upon completion of the Aupossom run, output files were loaded on Denroscope (version 3.5.7) for result visualization^37^. Visualization of the identified lead compounds based on the chemical interaction groupings by dendroscope was achieved using the PyMOL molecular visualizer^38^. All observed biomolecular interaction types were viewed using both the Protein Ligand Interaction Profiler^39^ and the MolADI tools^40^.

### Assessment of drug-likeness profile

The ADMET profile of selected lead compounds in comparison with the reference compound (masitinib) was estimated using a combination of the SwissADME^41^ and Pro Tox-II^42^. For both tools, the SMILES strings of each compound to be analyzed were used as input. The SwissADME was used for the evaluation of essential ADME parameters such as the physicochemical properties, lipophilicity, water solubility, pharmacokinetics, and drug likeness of the lead compounds, while the usage of the Pro Tox II was directed at the prediction of organ toxicity and toxicity endpoints such as hepatotoxicity, carcinogenicity, mutagenicity, and cytotoxicity. The oral toxicity of each lead compound was also estimated using Pro Tox II.

### Molecular dynamics simulation and post-simulation analysis

The molecular dynamics simulation was performed using GROMACS (version 2020)^43^. Poses of selected lead compounds from the Aupossom output were chosen as the simulation’s starting conformation. The Charmm36 forcefield^44^ was used for both SARS-CoV-2 3CLpro (apo) simulation and the simulation of its complex with the selected lead compounds, including the co-crystallized ligand (masitinib). The Cgenff web server^45^ was used for the generation of topology files for each ligand, while the system was solvated in a cubic water box using the TIP3P water model. Chloride ions were added for the neutralization of the systems, and energy minimization was performed with the application of position restraints for 50,000 steps. The system equilibration was also performed for 50,000 steps while system temperature and pressure were maintained at 300 K and 1 bar, respectively, followed by a 300-ns production run on all systems. Using the system trajectory files, we conducted several post-simulation analyses, such as the RMSD (root mean square deviation), RMSF (root mean square fluctuation), Rg (radius of gyration), SASA (solvent-accessible surface area), hydrogen bond analysis, PCA (principal component analysis), and the FEL (free energy landscape), in order to gain more insight into the differential system stability and dynamics upon ligand binding. The trajectory graphs were plotted using the XMGRACE software^46^, while visualization was carried out on the PyMOL molecular visualizer^38^.

### Target prediction

To evaluate the most probable set of macromolecular protein targets for each lead compound, we conducted a target prediction study using the SwissTargetPrediction tool^47^. This was necessary in order to identify from the lead compounds those that are less likely to inhibit tyrosine kinases, thereby greatly reducing the likelihood of possessing such tyrosine kinase inhibition-linked side effects as have been attributed to masitinib^23^.

## Results

### Unique co-crystallized masitinib pharmacophore-matching compounds exist across chemical databases

Drayman et al.^23^ in their recent work have recommended the development of analogs of masitinib with better inhibitory activity against the SARS-CoV-2 3CLpro and likewise possessing a lower degree of tyrosine kinase inhibition-associated side effects. More recently, Gurung et al.^48^ carried out a computational study to examine the toxicity and drug-likeness of various chemical moieties that were substituted for the N-methylpiperazine group of masitinib. The filtered analogues were put through molecular docking studies, and it was discovered that the analogues with the substituents morpholine in M23 (CID59789397), 4-methylmorpholine in M32 (CID143003625), and methylamine in M10 (CID10409602), have a stronger affinity to the 3CLpro than masitinib^48^.

**Figure 1:**
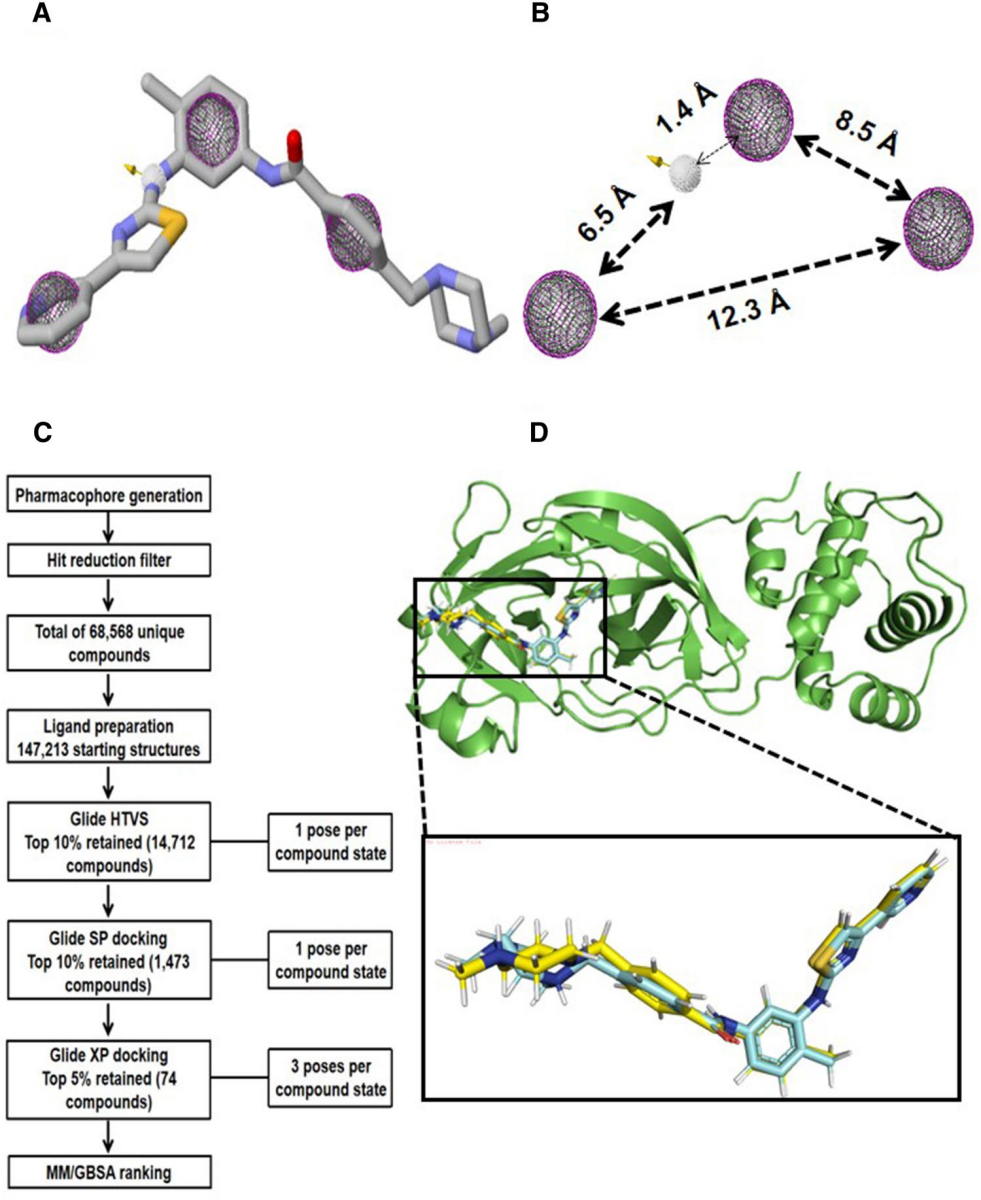
Pharmacophore modelling and virtual screening workflow. (**A**) The 3CLpro-bound pose of masitinib with selected pharmacophore features for chemical database screening. (**B**) The four selected pharmacophore points with individual distances. The single hydrogen bond donor is shown in a white color mesh, while the aromatic and hydrophobic features (mapped on each other) are shown in purple and green color meshes, respectively. (**C**) A workflow for the virtual screening protocol, starting from the pharmacophore generation stage to the final MM-GBSA ranking. (**D**) Zoomed-in superimposition of the docked masitinib 3D structure (represented in yellow stick format) against the 3CLpro co-crystallized masitinib structure (represented in cyan stick format) for the reliability test of the virtual screening protocol.

In an attempt to generate not just structural analogs but compounds that can mimic the binding pose and interaction profile of masitinib in the SARS-CoV-2 active site, we extracted the bound pose of the drug as co-crystallized with the 3CLpro, from which the pharmacophore features were selected^23^. Pharmacophore features on selected atoms of masitinib that make important biomolecular interactions with pocket residues of the SARS-CoV-2 3CLpro were selected on the assumption that compounds possessing the same set of features at similar distances are likely to exhibit a similar inhibitory effect. The selected pharmacophore features for screening include a hydrogen bond donor and three aromatic and hydrophobic features each (Fig. 1A,B). Having identified the pharmacophore-matching features, the several chemical compound databases screened returned a cumulative of 91,285, of which some are overlapping (Table 1). After we ran the in-house Python script to identify multiple recurrences of a single compound and remove redundancy^49^, a total of 68,568 unique compounds were retained. These sets of compounds served as the input structures for the ligand preparation process. The workflow for the virtual screening protocol begins with the pharmacophore generation and ends with the MM-GBSA ranking (Fig. 1C). The output of the docked masitinib structure with SARS-CoV-2 3CLpro maintained a reliably high pose comparable to the co-crystallized pose of masitinib with SARS-CoV-2 3CLpro (Fig. 1D)^50^.

**Table 1:** Number of pharmacophore-matching compounds identified per database screened.

Chemical databases screened	Number of generated pharmacophore-matching compounds
CHEMBL30	3003
ChemDiv	3728
ChemSpace	9428
MCULE	30,283
MCULE-ULTIMATE	21,373
MolPort	8781
NCI Open Chemistry Repository	8
WuXi LabNetwork	3495
ZINC	11,186
Cumulative	91,285

### MM-GBSA-based scoring identified and ranked lead compounds

As represented in the virtual screening workflow (Fig. 1C), a total of 74 compounds (including differential conformational states) were ranked as the final top hits based on the MM-GBSA pose reranking scoring function. The MM-GBSA scoring function has been optimized for binding free energy prediction with regard to a series of congeneric molecules and is therefore usually used after the completion of the glide docking/virtual screening workflow. Furthermore, ligand ranking based on MM-GBSA binding energy calculation can be expected to be highly consistent with experimental binding energy estimation, mostly when considering congeneric sets of molecules. The lower the estimated MM-GBSA binding energy value, the stronger the binding affinity^51^. However, we have selected the top 10 hits from the final output of the MM-GBSA ranking for further analysis in this study (Table 2, Supplementary Figs. 1A–J).

**Table 2:** List of the top 10 hits according to the MM-GBSA binding energy ranking in descending order.

Compounds	Prime energy	Docking score (Kcal/mol)	XP GScore	Glide gscore (Kcal/mol)	Glide emodel	MM-GBSA (Kcal/mol)
MCULE-1361639875	− 13,614.98	− 7.893	− 8.35	− 8.35	− 94.286	− 88.49
CHEMBL230286	− 13,678.65	− 9.476	− 9.476	− 9.476	− 100.305	− 84.4
CHEMBL3805890	− 13,616.78	− 9.446	− 9.446	− 9.446	− 90.924	− 82.09
CSC097356065	− 13,588.21	− 8.225	− 8.225	− 8.225	− 87.663	− 80.94
MCULE-6370590876	− 13,612.52	− 7.905	− 7.913	− 7.913	− 82.884	− 80.75
519959077	− 13,602.45	− 8.229	− 8.245	− 8.245	− 101.481	− 80.39
MCULE-6357305429	− 13,579.22	− 8.112	− 8.112	− 8.112	− 78.572	− 79.88
MCULE-8253455899	− 13,596.91	− 8.068	− 8.526	− 8.526	− 90.643	− 79.64
CHEMBL3642843	− 13,691.7	− 8.221	− 8.221	− 8.221	− 100.687	− 79.4
CHEMBL2058939	− 13,634.06	− 7.899	− 7.899	− 7.899	− 90.546	− 79.31

**Figure 2:**
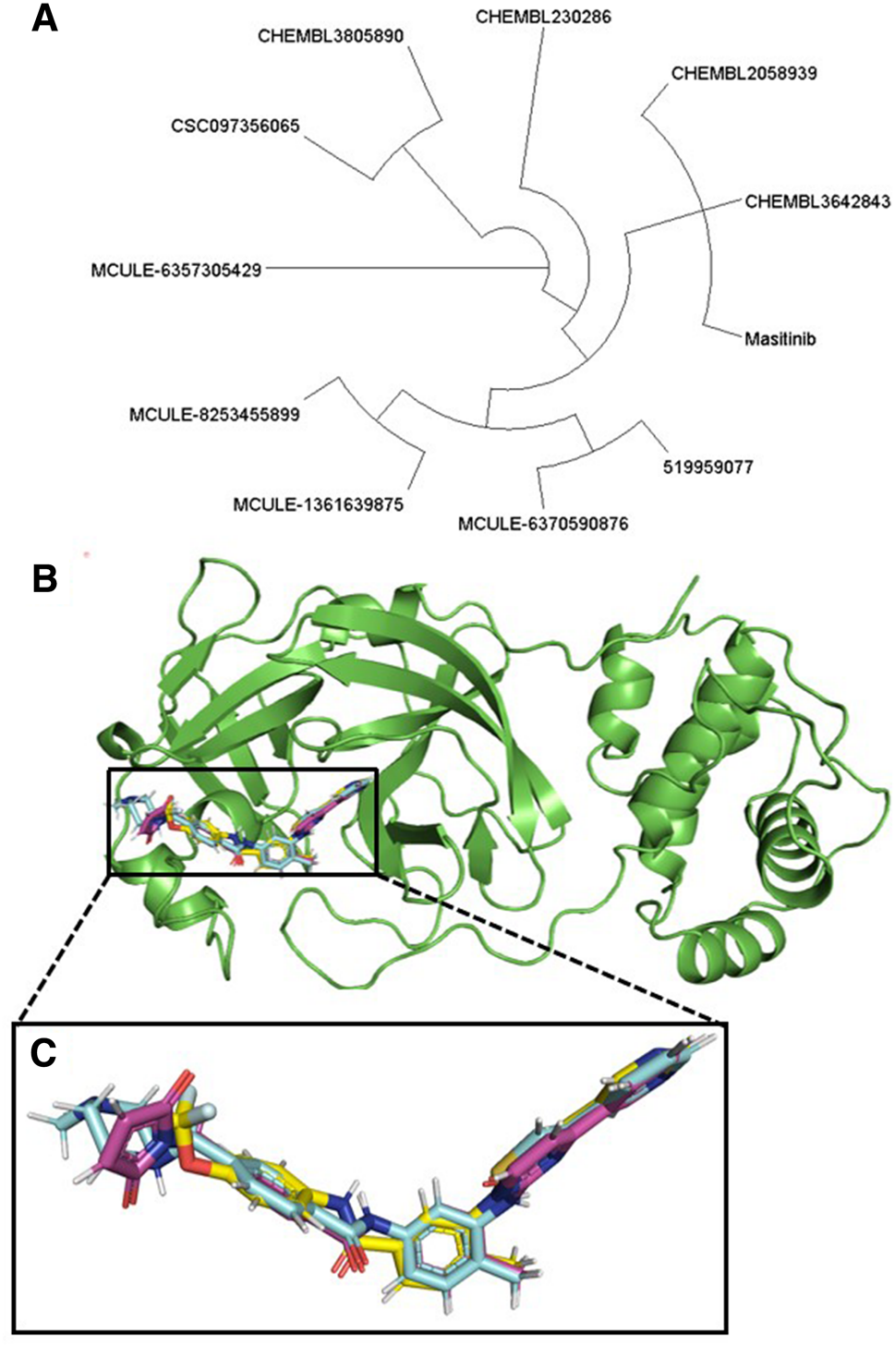
Lead compound identification through protein–ligand interaction fingerprinting. (**A**) AuPosSOM-calculated dendrogram for the top 10 hits of the MM-GBSA binding energy ranking and masitinib. The image was generated with the Dendroscope software. (**B**) The binding of masitinib, CHEMBL3642843, and CHEMBL2058939 to the SARS-CoV-2 3CLpro active site. (**C**) A zoomed-in view of the binding pose similarity between masitinib, CHEMBL3642843, and CHEMBL2058939 based on the protein–ligand interaction fingerprinting grouping. Masitinib is shown in cyan, while CHEMBL3642843 and CHEMBL2058939 are displayed in purple and yellow, respectively.

For the identification of the most promising set of compounds to be further analyzed, the protein–ligand interaction fingerprint of the top 10 hits from Table 2, along with masitinib, was generated using the AuPosSOM program. AuPosSOM evaluates protein–ligand complexes via existing atomic interactions and the use of self-organizing contact maps. In this case, the 3CLpro is the receptor, while the ligands to be evaluated are the top 10 hits from the MM-GBSA ranking, along with the co-crystallized masitinib^52^. Clustering in the self-organizing contact map is based on the degree of similarity in interactions between each evaluated ligand and the binding pocket residues of the receptor^52^. Using the Dendroscope program^37^, the generated dendrogram by Aupossom, which represents the various shared chemical interactions among the top 10 hits and masitinib, was visualized (Fig. 2A). The dendrogram gave comparative details on how each structural analog of masitinib in the top 10 hits shared their chemical interactions with one or multiple regions of the 3CLpro active site. Thus, each branch represents similarity groupings of the potential inhibitors based on both bound and unbound interactions with the active site residues of the SARS-CoV-2 3CLpro (Fig. 2A). The output suggests CHEMBL3642843 and CHEMBL2058939 might share the most similar interaction profile with masitinib, as these three fall within the same clade. A view of the binding poses of these compounds in the 3CLpro active site also strongly supports the AuPosSOM output (Fig. 2B,C). We therefore selected CHEMBL3642843 and CHEMBL2058939 along with masitinib for further analysis.

### Protein–ligand profiling revealed interaction profile of the lead compounds comparable to 3CLpro-masitinb complex

**Figure 3:**
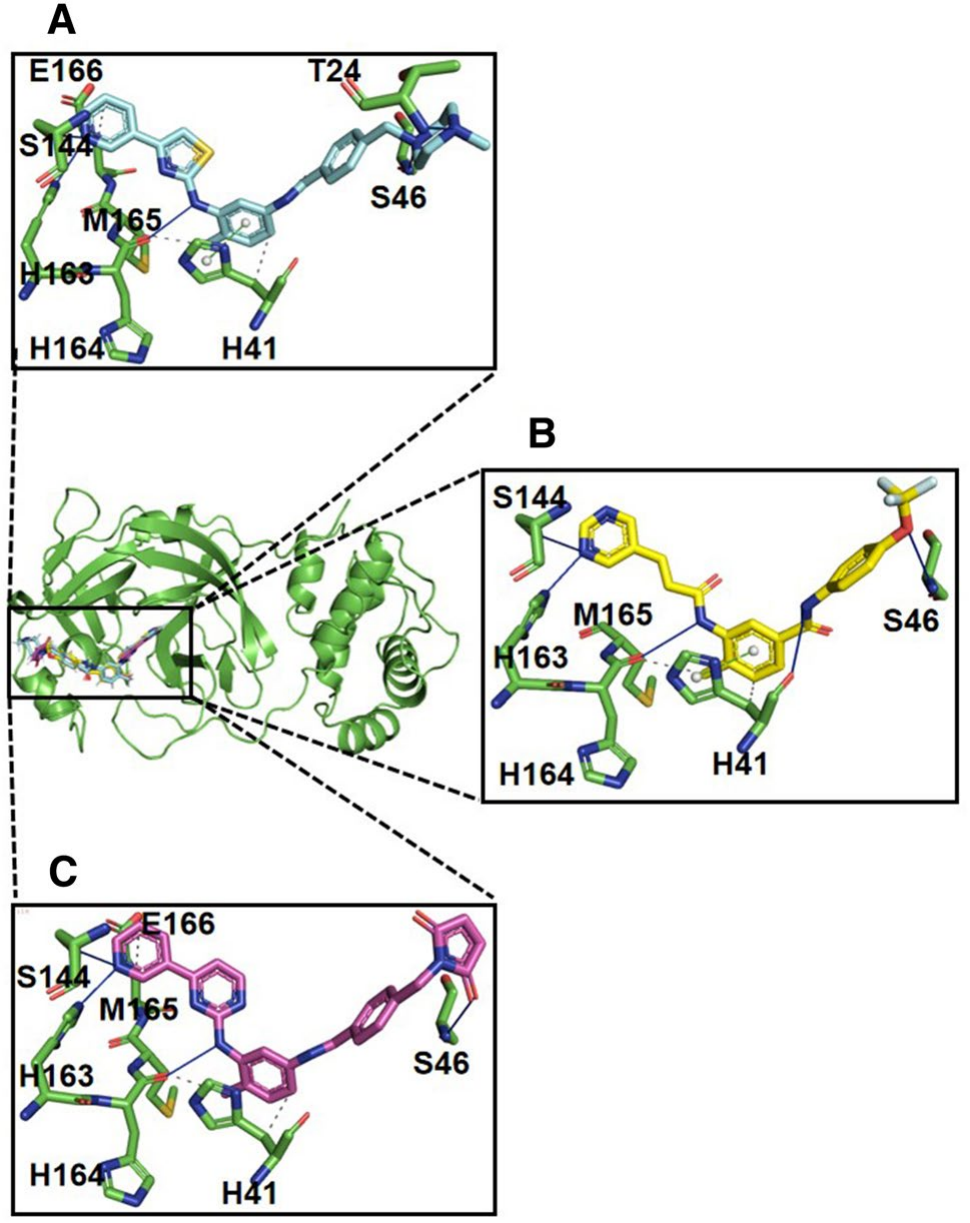
Protein–ligand interaction profile prediction of masitinib and the selected lead compounds. (**A**) The predicted interaction profile of masitinib in complex with the SARS-CoV-2 3CLpro. Masitinib is displayed in cyan-colored stick while interacting active site residues of the 3CLpro are displayed in green-colored sticks. (**B**, **C**) The predicted interaction profiles of CHEMBL2058939 (yellow stick) and CHEMBL3642843 (purple stick) respectively, after docking against the 3CLpro active site. All interacting active site residues are shown in green sticks.

Aiming to further delineate the interaction profile of the selected two lead compounds, the protein–ligand interaction profiler was used for the visualization of the interaction of the lead compounds with active site residues of the SARS-CoV-2 3CLpro (Fig. 3A–C). The structural description of the binding of masitinib to the SARS-CoV-2 3CLpro suggests that it interacts non-covalently between the I and II domains in order to block major catalytic residues at the two dimeric active sites^23^. Drayman et al.^23^ demonstrated that the pyridine ring of masitinib specifically fits into the S1 peptide recognition pocket of the 3CLpro. Aside from Van der Waals and hydrophobic interactions between the surrounding residues and the pyridine ring of masitinib, a hydrogen bond within a distance of 2.78 Å was also identified between the S1 pocket His163 and the pyridine ring. Two additional hydrogen bonds were identified between the aminothiazole ring of masitinib and the 3CLpro residues, with one formed between His164 and the amine group of masitinib at a distance of 3.09 Å and the other between Cys145 (a crucial 3CLpro catalytic residue) Sγ atom and masitinib’s thiazole nitrogen at a distance of 3.38 Å. Furthermore, a perfect π–π stacking interaction was shown to be formed between His41 (the second residue making up the catalytic dyad of the protease) and masitinib’s hydrophobic toluene ring while occupying the protease S2 binding cavity, and the benzamide also shown to interact mainly with the protease main chain between Cys44 and Ser46^23^.

Aside from the absence of the previously described interaction between Cys145 and the thiazole nitrogen of masitinib in our predicted interaction profile for the 3CLpro-masitinib complex (Fig. 3A), the protein–ligand interaction profiler accurately identified other major existing interactions consistently with the earlier report, suggesting a high level of confidence in the prediction output for each protein–ligand interaction profile. Furthermore, consistent with the protein–ligand interaction fingerprinting result (Fig. 2A,B), the interaction profiles of CHEMBL3642843 and CHEMBL2058939 in complex with the 3CLpro (Fig. 3B,C) showed great similarity with the predicted interaction profile of the 3CLpro-masitinb complex (Fig. 3A). Another important factor to note in the structure of CHEMBL2058939 is the presence of a double bond, which is expected to have an impact on the binding conformation and activity of the compound as it entails more than one geometry (Supplementary Figs. 1, 2). However, the docked pose to the catalytic pocket of the SAR-CoV-2 3CLpro suggests that the active state of the compound might be the Trans isometric geometry (Supplementary Fig. 2C).

### The lead compounds exhibit acceptable drug properties based on ADMET evaluation

Structural analysis of the selected chemical structures suggests that both prospective lead compounds (CHEMBL3642843 and CHEMBL2058939) contain reactive moieties such as vinyl Michael acceptor 1 and maleimide in CHEMBL3642843 (Supplementary Fig. 3A,B). Via S-Michael addition reactions, maleimide is frequently used to conjugate with thiol groups. The resultant thiolated maleimide is frequently utilized as a helpful handle in chemical synthesis and exhibits distinct biological activity^53^. Maleimide is also frequently employed to make antibody–drug conjugates (ADCs), although this method can result in drug loss in the bloodstream due to a retro-Michael reaction, as seen in Adcetris^54^. Analysis of the CHEMBL2058939 chemical structure also shows it contains a Michael acceptor (Supplementary Fig. 3C). Being electrophilic agents, Michael acceptors may create covalent connections to biological organisms' DNA and nucleophilic protein sites, causing disorders such as allergic contact dermatitis, carcinogenicity, and excess toxicity (as contrasted to unspecific narcosis or baseline toxicity) for aquatic creatures. Additionally, cyclopentenone prostaglandins' anti-inflammatory activity, the activation of nerve system ion channels, the induction of enzymes guarding against carcinogenesis, pathologies connected to atherosclerosis and oxidative stress, and the Michael addition to endogenous cysteine thiol sites are all affected^55^.

To evaluate the ADMET profile of the selected lead compounds, we used an in silico approach, which has been widely used because of its capacity to save time and cost in the process of drug development^56^. ADME properties were estimated using the SwissADME web server^41^, while the toxicity profile was estimated using the Pro Tox-II server^42^. The ADMET analysis in this study covered the evaluation of the physicochemical properties, lipophilicity, water solubility, pharmacokinetics, drug-likeness, medicinal chemistry properties, oral toxicity, and toxicity endpoint of each selected lead compound and masitinib (Supplementary Tables 1–7, Supplementary Figs. 4A–C, 5). The analysis includes individual parameters such as, the calculation of number of heavy atoms, number of hydrogen bond donors and acceptors, number of aromatic heavy atoms, fraction Csp3 (ratio of sp^3^ hybridized carbons over the total carbon count of the molecule), molar refractivity, topological polar surface area, consensus Log P, and Log S estimation using several water solubility prediction models. Parameters such as gastrointestinal absorption, blood–brain barrier permeation, substrate/inhibitor of the P-glycoprotein, and the cytochrome P450 protein family were also estimated alongside the evaluation of drug-likeness and medicinal chemistry parameters with different prediction models. Finally, individual toxicity parameters such as the lethal dose, toxicity class, hepatotoxicity, carcinogenicity, immunotoxicity, mutagenicity, and cytotoxicity of individual lead candidates were evaluated. The analyses taken together suggest that both masitinib and the identified lead compounds possess acceptable ADMET properties for good drug candidates, and as such, they were analyzed further for activity against the SARS-CoV-2 3CLpro (Supplementary Tables 1–7).

### MasitinibL binding alters the molecular trajectory of 3CLpro

**Figure 4:**
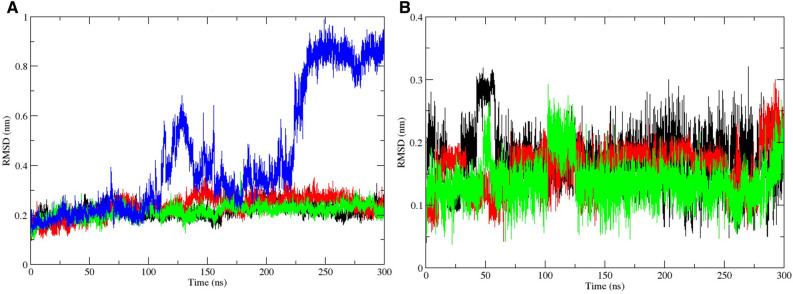
Root mean square deviation plots from molecular dynamics simulation. (**A**) represent the RMSD plot for the 3CLpro C-alpha backbone. Trajectories for the Apo, and complexes with masitinib, CHEMBL3642843 and CHEMBL2058939 are shown in black, red, green, and blue colors respectively. (**B**) The ligand RMSD plot. Trajectories for masitinib, CHEMBL3642843 and CHEMBL2058939 are shown in black, red, and green colors, respectively.

The molecular dynamics simulation study was carried out in order to evaluate the effect of ligand binding on the dynamics and stability profile of the SARS-CoV-2 3CLpro. By computing the root mean square deviation based on the atomic backbone of both the apo and inhibitor-bound 3CLpro, the equilibrium state of the protein was evaluated comparatively. Root mean square deviation is used for the measurement of changes in the structural backbone of proteins between the initial and final conformational states. Protein stability relative to conformation can be evaluated through the trajectory deviations during simulations. The greater the deviation, the more unstable a protein is likely to be^57^. We estimated the changes in the stability profile of the 3CLpro both in the unbound (apo) state and the ligand-bound state. The RMSD plot over 300 ns of simulation time, as shown in Fig. 4A, suggests that CHEMBL2058939 might be a very potent inhibitor of 3CLpro. Although no significant differences were observed in the root mean square deviation of each trajectory as of 100 ns, However, a highly significant rise in RMSD was observed for the CHEMBL2058939-bound 3CLpro trajectory after 100 ns of simulation time, and another sharp rise in the RMSD was observed at about 230 ns of simulation time to hit a peak of about 0.9 nm. This particular deviation was massive compared to the maximum deviations generated by the other systems (including the masitinib-bound 3CLpro trajectory) (Fig. 4A, Supplementary Fig. 6A–C). This signifies that the binding of CHEMBL2058939 to the 3CLpro active site is likely to result in a huge conformational instability in the viral protein.

We also estimated the root mean square deviation of each 3CLpro-bound ligand. Although significant fluctuations were observed in the RMSD trajectory of each bound ligand at different stages during the simulation period, signifying a state of instability at those points. However, the trajectories were stabilized before 150 ns, and the stability was maintained till the end of the simulation time for each ligand. The root mean square deviation value of CHEMBL2058939 upon binding the 3CLpro active site was also observed to be maintained at about 0.1 angstroms for the most part of the period of simulation (the lowest), suggesting that CHEMBL2058939, in comparison to the other 3CLpro-bound ligands, might be the most stable upon receptor binding (Fig. 4B).

**Figure 5:**
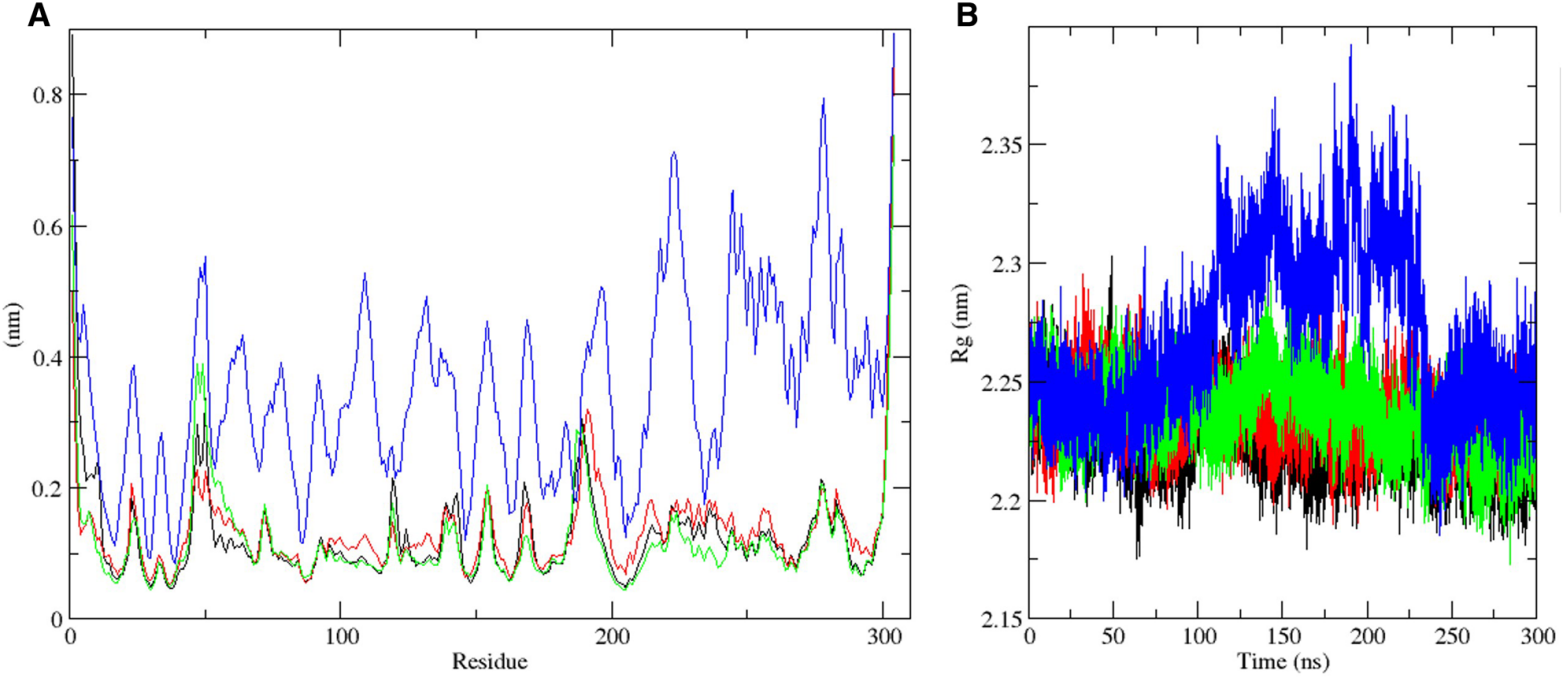
Root mean square fluctuation and radius of gyration plots. (**A**) The RMSF plot for the 3CLpro structural backbone. The 3CLpro apo trajectory and trajectories of its complexes with masitinib, CHEMBL3642843 and CHEMBL2058939 are displayed in black, red, green, and blue colors respectively. (**B**) The radius of gyration plot from the 300 ns molecular dynamics simulation run. Trajectories of the apo protein and its complexes with masitinib, CHEMBL3642843 and CHEMBL2058939 are displayed in black, red, green, and blue colors respectively.

Structural mobility analysis and molecular dynamics simulation play crucial roles in the interpretation of data, mostly for biomolecules. The most frequently used measures of mobility that are computed from molecular dynamics simulations are the structural root mean square deviation and root mean square fluctuation. Both factors are estimated after atomic coordinate alignment in trajectory steps to a reference structure^58,59^. The root mean square fluctuation specifically provides a measure of an average particle deviation (deviation of individual residues of proteins) from a reference point over a particular period of time. Thus, the root mean square deviation analyzes structural portions that fluctuate the most or the least from their mean structure^60^. In comparison with the other analyzed systems, the highest residue fluctuation was observed in the trajectory representing the 3CLpro-CHEMBL2058939 complex (Fig. 5A, Supplementary Fig. 7A–C). This shows great consistency with the root mean square deviation profile of CHEMBL2058939, which upon binding the SARS-CoV-2 3CLpro active site resulted in a massive increase in trajectory fluctuation (Fig. 4A, Supplementary Fig. 6A–C).

The radius of gyration of a protein represents the distribution of its atoms around its axis. The length, showing the distance between the rotation point and the point where energy transfer exerts its maximum impact, gives the radius of gyration. This concept further helps in the identification of different types of polymers (as in the case of a protein). Distance calculations and the estimation of the radius of gyration are among the most important and widely used indicators for the prediction of macromolecular structural activities^61^. Upon the binding of lead compounds/ligands, proteins experience changes in conformation that alter their radius of gyration^62^. Protein structural compactness is directly related to the folding rate, which can be monitored via the radius of gyration calculation. A good comprehension of the radius of gyration further helps in the prediction of the binding patterns and compactness of proteins and drugs^62^.

In order to evaluate the changes in the degree of compactness of the SARS-CoV-2 3CLpro upon ligand binding, we analyzed the radius of gyration trajectories after a 300 ns molecular dynamics simulation period. The high degree of fluctuation observed in the trajectory representing the 3CLpro-CHEMBL2058939 complex suggests that the viral protein exhibited the lowest degree of compactness upon the binding of the lead compound (Fig. 5B, Supplementary Fig. 8A–C). This further increases the confidence in the outcomes of previous analyses, all of which have suggested a high level of structural instability upon the binding of CHEMBL2058939.

**Figure 6:**
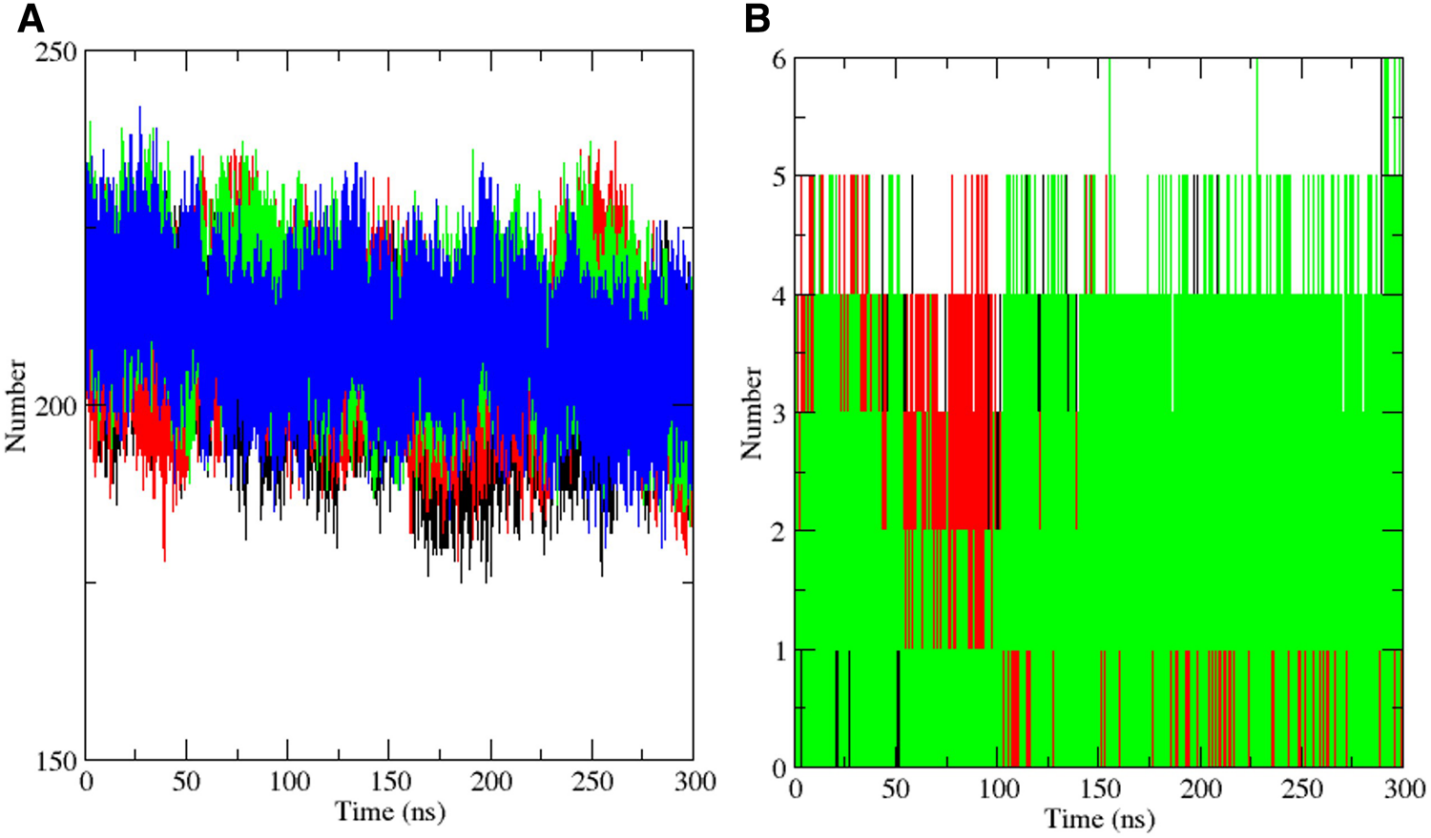
Hydrogen bond plots from molecular dynamics simulation. (**A**) The intra-protein H-BOND analysis plot of the SARS-CoV-2 3CLpro. The apo protein H-BOND plot and the plot of its complexes with masitinib, CHEMBL3642843 and CHEMBL2058939 are shown in black, red, green, and blue, respectively. (**B**) The protein–ligand H-BOND plot over 300 ns of simulation time. Trajectories for masitinib, CHEMBL3642843 and CHEMBL2058939 are shown in black, red, and green, respectively.

Hydrogen bonds have justifiably been known as the master key of molecular recognition. Because hydrogen bonds have a stronger interaction than van der Waals interactions but a weaker interaction than covalent bonds, their flexibility and ubiquity make them the most important biomolecular system physical interaction in aqueous solution. Hydrogen bonding plays critical roles in several biological and chemical processes, including enzyme catalysis and ligand binding^63,64^. Analysis of the intra-protein hydrogen bonding of the SAR-CoV-2 3CLpro suggests that the binding of each compound has less impact on the hydrogen bond profile of the viral protein, as there were no significant differences between the trajectories of the apo protein and its complex with the lead compounds and masitinib (Fig. 6A, Supplementary Fig. 9A–C). Analysis of the protein–ligand hydrogen bond interaction trajectory showed that a total of six hydrogen bond interactions were formed between the complex involving the 3CLpro and CHEMBL2058939 (Fig. 6B). However, five of these interactions were predominantly constant all through the period of simulation, unlike the other analyzed systems where a total of five hydrogen bond interactions were observed and much fewer were constant throughout the period of simulation (Fig. 6B). Consistent with the ligand RMSD analysis, this further suggests that CHEMBL2058939 might form a more stable complex with the SARS-CoV-2 3CLpro upon binding to the active site.

**Figure 7:**
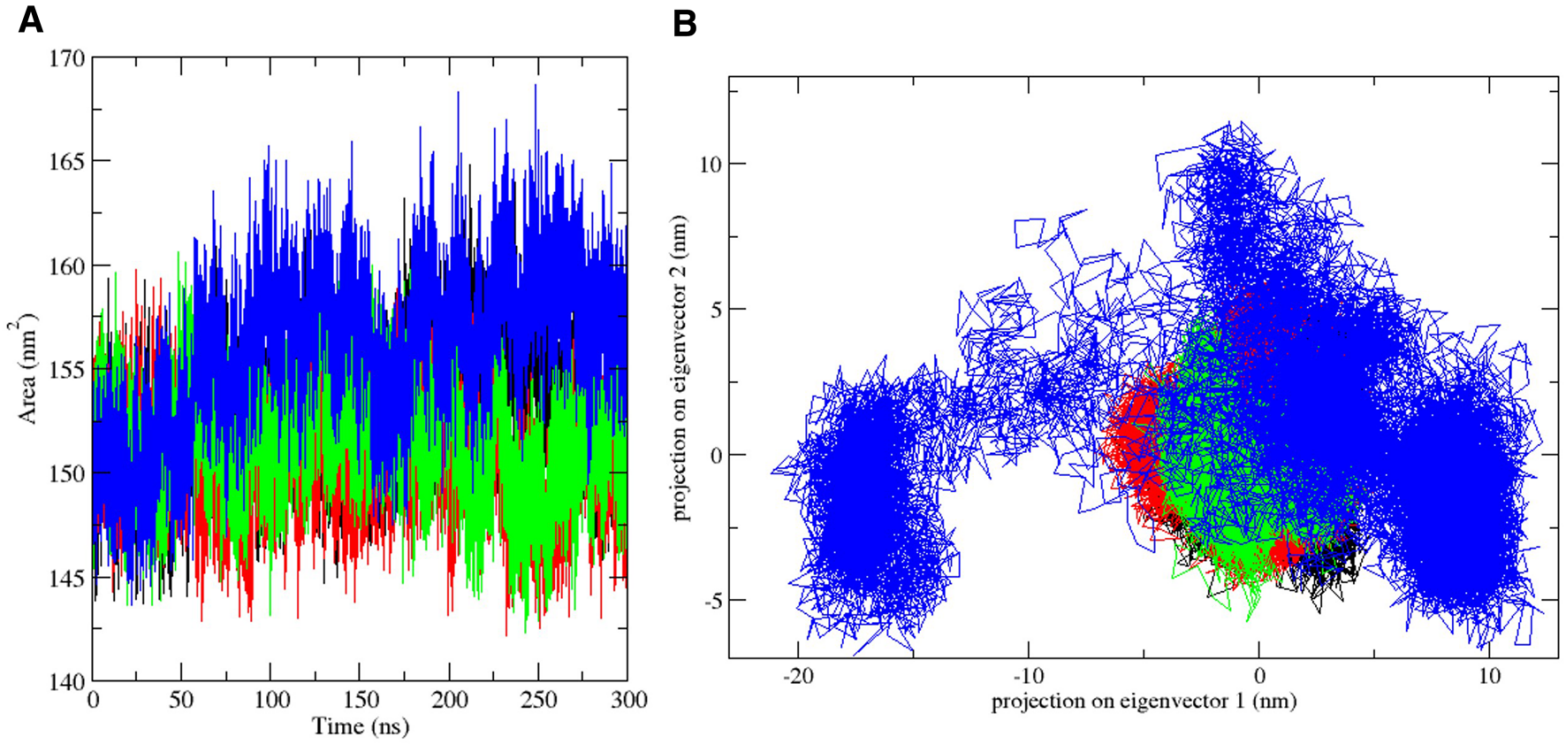
Solvent accessible surface area and principal component analysis plots. (**A**) represents the plot of solvent accessibility for the apo protein and its complexes with masitinib, CHEMBL3642843 and CHEMBL2058939. Their individual trajectories are shown in black, red, green, and blue, respectively. (**B**) A 2-dimensional projection of the principal components of each system. The collective motion trajectory of the apo protein and its complex with masitinib, CHEMBL3642843 and CHEMBL2058939 are displayed in black, red, green, and blue, respectively.

Lee and Richards introduced the solvent accessibility concept for globular protein residues in 1971. It was defined as the extent to which surface atoms of proteins can make contacts with a solvent. In biological systems, the solvent is taken as water with a 1.4 Å radius. This represents the locus of the center of the solvent molecule as it rolls along the protein, making the maximum allowed van der Waals interactions without penetrating other atoms. Lee and Richards have also described it as the "static accessible surface area," since potential flexibility was excluded from the system^65^. Solvent accessible surface area (SASA) is a quantity of great interest in functional studies and protein folding, as it plays a crucial role in the delineation of the structure–function relationship of proteins. The burying of hydrophobic residues is an essential factor for the folding of proteins. Therefore, the exposure of these residues to the hydrophobic core and solvent is directly related to protein stability^4,66^. The solvent-accessible surface area analysis was carried out over the 300-ns simulation period in order to evaluate the changes in the stability profile of the SARS-CoV-2 3CLpro upon the binding of potential lead compounds and the co-crystallized masitinib. The trajectories of the other systems, in comparison with the apo, have displayed increases in the solvent accessible surface area of the viral protein at varying degrees from the start of the simulation. However, the generated trajectory from the SASA plot showed that the complex of CHEMBL2058939 with the SARS-CoV-2 3CLpro resulted in the largest surface area exposure all through the period of simulation, likewise suggesting a decrease in the viral protein stability upon the binding of the potential lead compound (Fig. 7A, Supplementary Fig. 10A–C).

Protein structures and structures of other biological macromolecules are characterized by a set of multidimensional variables, such as dihedral angles and atomic coordinates, and details with respect to the dynamics of these macromolecules are typically derived as experimentally determined structural ensembles or as a time series of high-dimensional data. Although the large ensemble of dimensional data is composed of essential details, such information is difficult to interpret. The extraction of crucial features from high-dimensional data is therefore important for the understanding of the dynamics of biological macromolecules^67^. The principal component analysis is used in many research areas for the reduction of dimensions in high dimensional datasets. Biological macromolecules demonstrate many degrees of freedom, which allows the adoption of intricate structures and the exhibition of complex functionalities. The principal component analysis decomposes the overall motion of biological macromolecules over a specific period of simulation into a mode set, which can be ordered from the largest to smallest contributions to the root mean square fluctuation of the protein as estimated by the eigenvalues of the covariance matrix^67^.

A 2D projection of the principal component trajectories (PC1 and PC2) was used for the evaluation of the collective motion of all the systems over a 300 ns simulation time (Fig. 7B, Supplementary Fig. 11A–C). The PCA plot showed that the system representing the SARS-CoV-2 3CLpro-CHEMBL2058939 complex occupied a much wider phase space compared to the apo and other systems. In principle, systems with collective motions occupying a lesser phase space and likewise forming regular clusters represent stable systems, while the distribution of collective motion over a wider phase space with irregular clusters suggests relative instability^68^. This shows great consistency with previous analyses to further suggest that the binding of CHEMBL2058939 to the active site of the 3CLpro might result in massive conformational instability in the viral protein.

**Figure 8:**
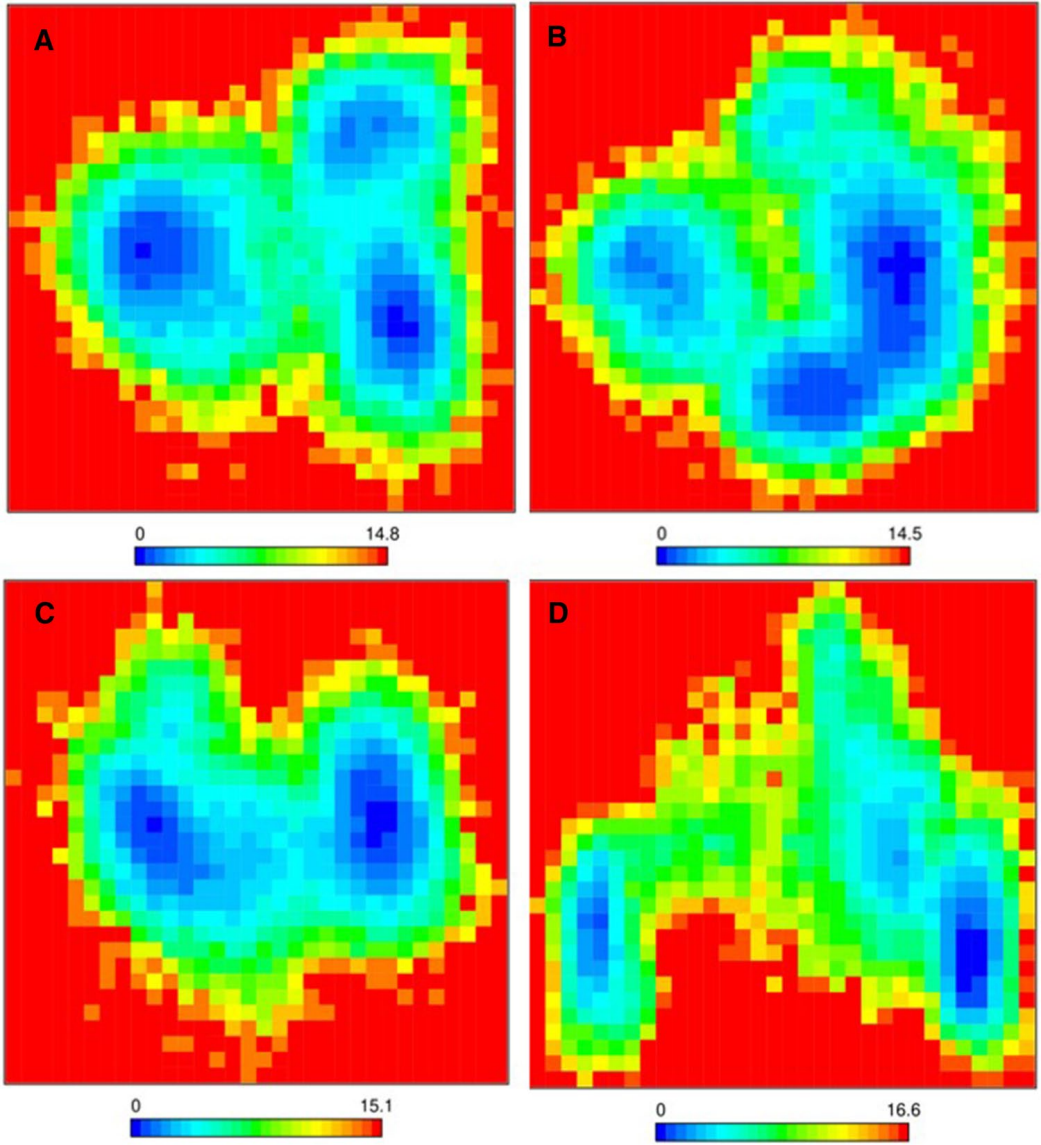
Free energy landscape (FEL) plots of the first two principal components. (**A**) Apo protein FEL plot, (**B**) FEL plot of the 3CLpro-masitinib complex, (**C**) FEL plot of the 3CLpro-CHEMBL3642843 complex, (**D**) FEL plot of the 3CLpro-CHEMBL2058939 complex.

An energy landscape depicts a map of different possible systemic states. The concept provides descriptions of all possible conformational states of a molecular system, the spatial positioning of interacting molecules, and their corresponding levels of energy (basically the Gibbs free energy)^69^. The free energy landscape is, geometrically, a plot of energy function across a systemic configuration space. While proteins can exist theoretically in a nearly limitless number of conformations along their energy landscape, in reality they relax into secondary and tertiary structural conformations with the lowest possible free energy^69^. The folding funnel hypothesis represents a major concept in the application of energy landscapes to the folding of proteins. The hypothesis is based on the assumption that the native state of a protein correlates with its free energy minimum under solution conditions regularly experienced in cells. Although free energy landscapes might tend to be rough with many non-native local minima that can trap partially folded proteins, the assumption of the folding funnel hypothesis likens the native state to a deep free energy minimum with steep walls that corresponds to a well-defined tertiary structure^70^. We evaluated the free energy landscape of each system in order to study the underlying conformational stability changes upon ligand binding to the SARS-CoV-2 3CLpro. The landscapes were extracted from PC1 and PC2 of the principal component analysis. The dark blue segment of each free energy landscape plot represents the most stable conformational state with the lowest energy for each system (Fig. 8A–D). As compared to the other systems, there exists a very high energy barrier between the three global minima generated in the free energy landscape plot representing the 3CLpro-CHEMBL2058939 complex, suggesting that the viral protein, upon binding the lead compound, underwent a massive conformational change as indicated by the energy barriers between each conformational basin (Fig. 8D). This also shows great consistency with all previous analyses to suggest that CHEMBL2058939 might be a potential lead compound for COVID-19 therapeutics.

### MasitinibL exhibit lower affinity for tyrosine kinases

**Figure 9:**
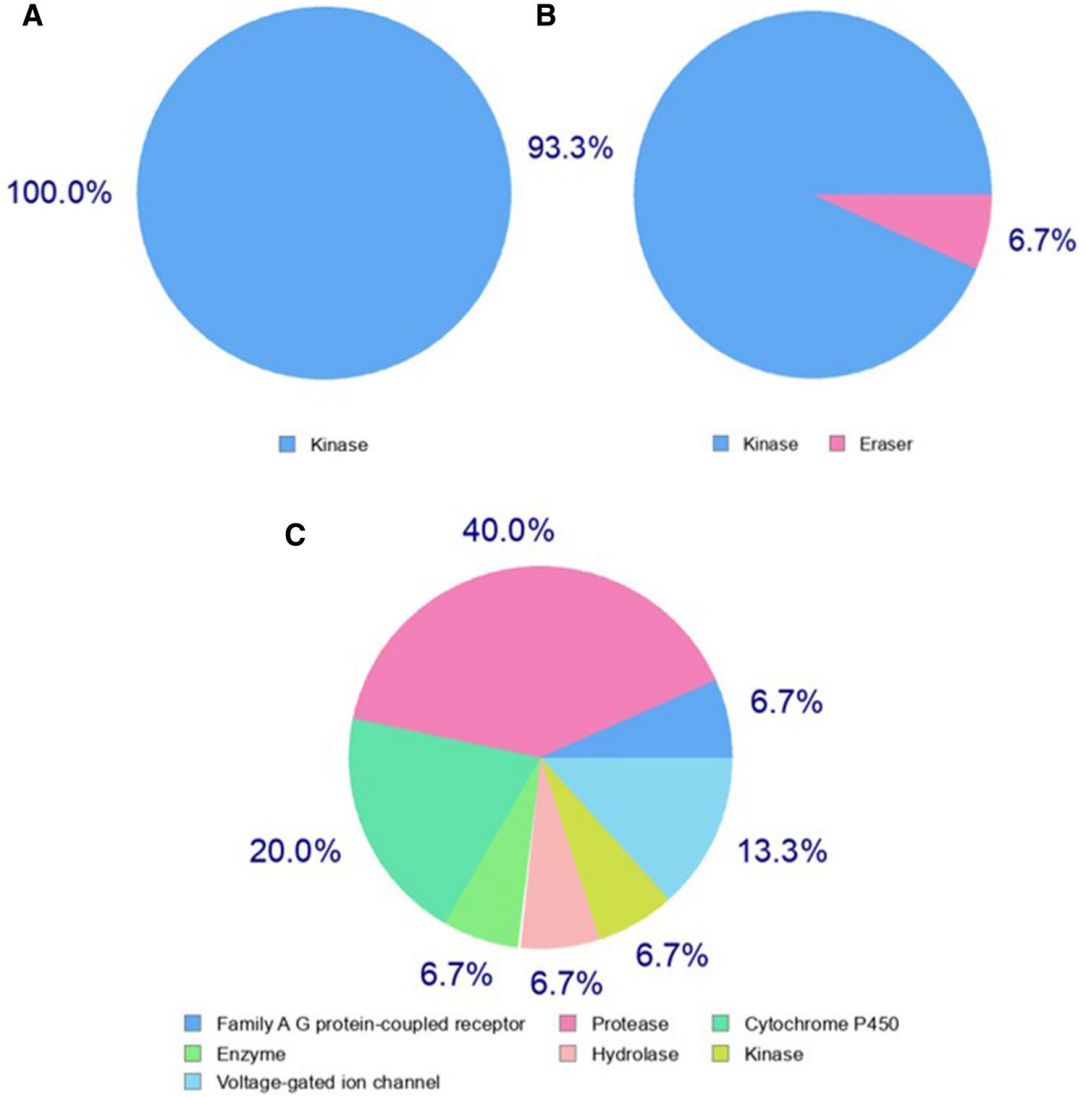
Pie chart showing the top 15 predicted target classes for each compound. (**A**) The predicted target class for masitinib. (**B**) The predicted classes of target for CHEMBL3642843. (**C**) The predicted classes of target for CHEMBL2058939.

While masitinib has been shown to display an excellent inhibitory profile against the SARS-CoV-2 3CLpro, the reported side effects as a result of its inhibitory activity against tyrosine kinases remain an issue of concern^23^. As a key objective in this study, we aimed to identify, among the selected lead compounds, analogs of masitinib with lesser anti-tyrosine kinase activity. To achieve this objective, we used the SwissTargetPrediction server, which predicts accurately targets of bioactive compounds centered on a combination of 2- and 3-dimensional measures of similarity with known ligands^47^. The prediction output was in excellent agreement with previous reports on the anti-tyrosine kinase activity of masitinib and showed that the drug is highly selective for kinase targets (Fig. 9A). Furthermore, seven tyrosine kinases were found among the list of predicted top 15 targets, including tyrosine-protein kinase LCK, tyrosine-protein kinase SRC, tyrosine-protein kinase FYN, tyrosine-protein kinase ABL, tyrosine-protein kinase YES, tyrosine-protein kinase BLK, and tyrosine-protein kinase Lyn (Supplementary Fig. 12). CHEMBL3642843 also demonstrated a high preference for kinases, with 93.3% of its predicted targets being kinases (Fig. 9B). Additionally, four tyrosine kinases were identified among its predicted top 15 classes of targets, including tyrosine-protein kinase ABL, tyrosine-protein kinase SYK, tyrosine-protein kinase receptor FLT3, and tyrosine-protein kinase LCK (Supplementary Fig. 13). However, the predictive model identified no single tyrosine kinase among the list of predicted top 15 targets of CHEMBL2058939 (Supplementary Fig. 14), even though a negligible percentage (6.7%) of its top class of predicted targets were identified as kinases (Fig. 9C). This strongly suggests that CHEMBL2058939 (hereafter named and also referred to as “masitinibL” or “masitinib-like”) might be the most suitable therapeutic analog of masitinib for the inhibition of the SARS-CoV-2 3CLpro.

Furthermore, the masitinibL (CHEMBL2058939) target prediction information as obtained from the ChEMBL database^71^ suggests that the compound might also be active against the human microtubule-associated protein 2 (CHEMBL2390810), with an activity threshold score of 6. The human herpes virus 5 capsid protein 40 (CHEMBL3771) also falls among the top predicted targets of masitinibL (likewise with an estimated activity threshold score of 6). The prediction method, which involves a detailed comparative study between the traditional quantitative structure–activity relationship (QSAR) and the Mondrian conformal prediction (MCP)^72^, further substantiates the initial prediction of the low preference of masitinibL for tyrosine kinases (Fig. 9).

### MasitinibL binds to 3CLpro through a variety of molecular bonds and interactions

**Figure 10:**
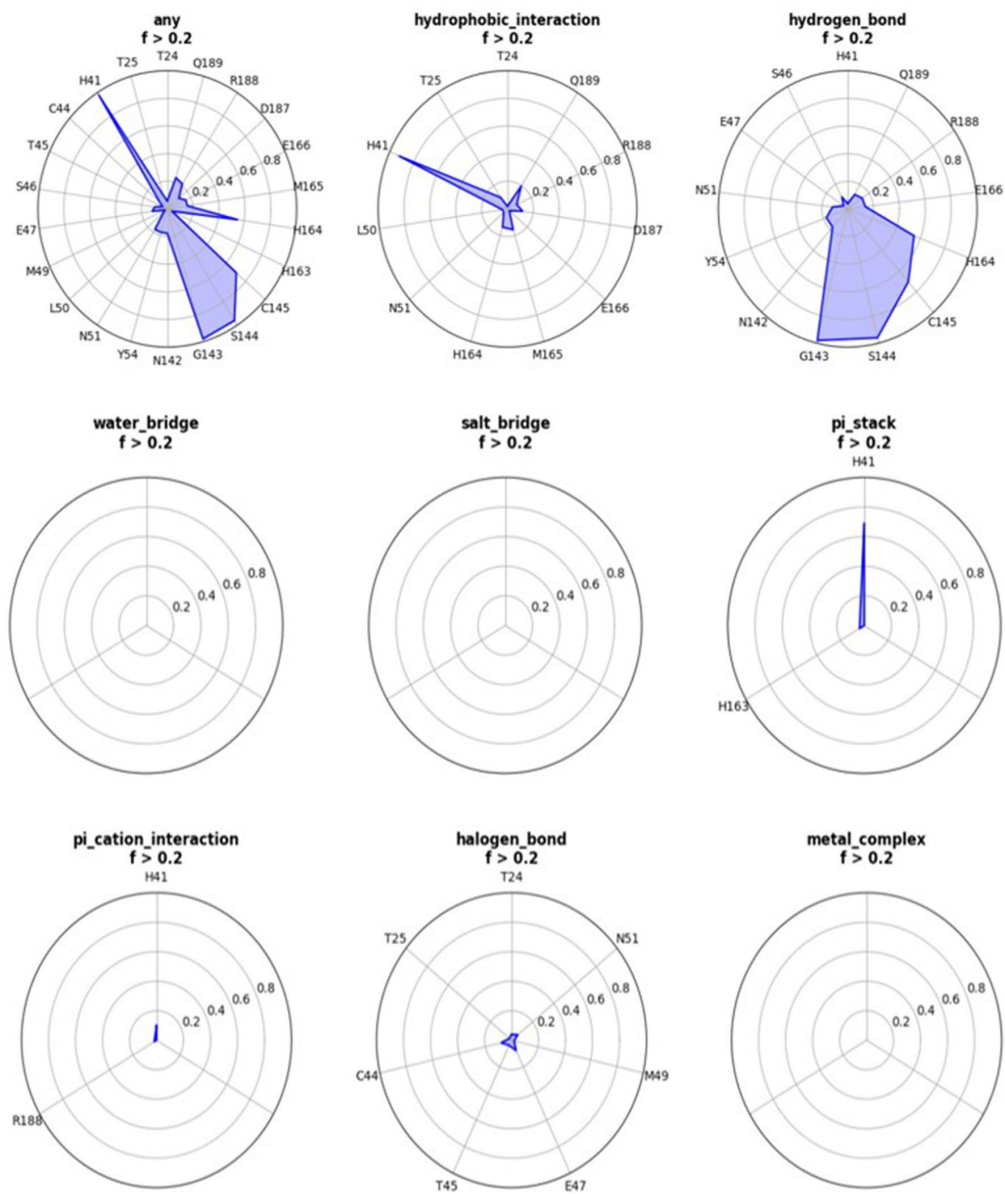
2D representation of the dynamic protein–ligand interaction profile of CHEMBL in complex with the SARS-CoV-2 3CLpro. All observed forms of interaction include hydrophobic interaction, hydrogen bond, pi stacking interaction, pi cation interaction, and halogen bond.

To delineate the interaction profile of masitinibL (CHEMBL2058939) in complex with the SARS-CoV-2 3CLpro over the final 50 ns of the simulation period, we subjected the 3-dimensional coordinate file and the trajectory file to a dynamic protein–ligand interaction analysis using the MolADI tool^40^. The dynamic interaction analysis was restricted to the final 50 ns of the simulation time as a result of the MolADI trajectory file size limitation (a maximum of 100 megabytes). Consistent with the reported interaction profile of masitinib with the active site residues of the SARS-CoV-2 3CLpro^23^, masitinibL maintained a strong hydrogen bond interaction with Cys145 and His164 all through the simulation period (Fig. 10, Supplementary Fig. 15). Furthermore, stronger hydrogen bond interactions were shown to exist between atoms of masitinibL and residues of the viral protein active site, such as Gly143 and Ser144 (Fig. 10, Supplementary Fig. 15). His41 in addition to the consistent pi stacking interaction with masitinibL, also formed a strong hydrophobic interaction and a weaker pi cation interaction. Other residues involved in weak hydrophobic interactions include His164, Met165, Asp187, and Gln189 (Fig. 10, Supplementary Fig. 15). Finally, several weak halogen bonds were observed between the lead compound and active site residues such as Thr24, Cys44, Glu47, and Asn51 (Fig. 10, Supplementary Fig. 15). Collectively, this analysis suggests essential interactions for efficacious inhibitory activity against the SARS-CoV-2 3CLpro.

## Discussion

SARS-CoV-2 infection remains a major health concern globally due to the resurfacing of different variants of the virus. Although several vaccines have received authorization for emergency use, and their deployment is currently ongoing worldwide, vaccinating the entire population of the world will take a long time^6^. New viral escape mutants might also likely emerge as a result of the slow rate of vaccination, eventually making vaccines ineffective^6,7^. The development of novel antiviral therapeutics and other broad therapies for possible future emergence is therefore of great necessity. One of the most effective strategies for designing therapeutics against SARS-CoV-2 is to target the core essential protein machinery required for the lifecycle of the virus. Upon infection, SARS-CoV-2 gains entrance into the cytoplasm of the host, where its genome is translated into about thirty different proteins. Sixteen of these are initially translated as two polyproteins that must undergo cleavage into individual proteins to facilitate the progression of infection. Two proteases encoded by the virus mediate this cleavage, and they include 3CLpro and PLpro^73,74^ and have become major hotspots for drug targets via de novo development or drug repurposing.

A recent drug repurposing screening study found that masitinib is a broad antiviral with high inhibitory activity against the 3CLpro of picorna- and coronaviruses and was also effective in treating mice infected with the SARS-CoV-2^23^. However, the study pointed out a main limitation of masitinib resulting from its high tyrosine kinase preference, which could be responsible for the side effects of masitinib. Hence, the study strongly recommended the development of therapeutics with comparable efficacy but lower tyrosine kinase activity. In light of this, we identified key pharmacophore features in the 3CLpro-bound pose of masitinib that make crucial biomolecular interactions with active site residues of the viral protein. From this set of pharmacophore features, we screened known chemical databases for compounds matching the model. With the aid of an extensive virtual screening workflow (HTVS, SP, and XP) and MM-GBSA pose ranking approach, top hits were identified as potential masitinib analogs.

One of the major challenges of the virtual screening approach was the identification of active compounds from a group of docked compounds, as the calculation of binding affinity in this method is typically based on scoring function estimation^75^. Scoring functions ideally should be able to identify the correct poses for each docked molecule and as well rank ligands according to their affinity for the receptor. However, the relevant binding affinity estimation for the ideal poses remain a challenging task^76^. We have, therefore, implemented the protein–ligand interaction fingerprinting method^52^ for the selection of potential lead compounds among the top 10 outputs of the MM-GBSA ranking (Table 2, Supplementary Fig. 1). Using the co-crystallized masitinib as reference, the constructed fingerprint map identified CHEMBL3642843 and CHEMBL2058939 (ranked 9th and 10th based on the MM-GBSA binding affinity estimation) in the same interaction fingerprint group as masitinib (Fig. 2A,B). This suggests that the two compounds share close protein–ligand interaction similarities with the co-crystallized masitinib and, as such, can be potential lead compounds for additional computational investigation. The suggested interaction similarities were further visualized using the protein–ligand interaction profiler (Fig. 3A–C).

To be an effective drug, a sufficient concentration of a compound must get to its cellular target and be retained long enough in its bioactive form for the required therapeutic activity to occur. The development of drugs involves ADMET (absorption, distribution, metabolism, excretion, and toxicity) assessment, but in cases where access to physical samples of the experimental compounds is not feasible, computational assessment remains an acceptable alternative for ADMET evaluation^77^. Additionally, different challenges, such as ethical concerns with regard to trials on animals, time, and cost, have limited the possibility of conducting experimental evaluations on potential therapeutic agents, thus making in silico assessment an inevitable approach for ADMET prediction^78^. Harnessing the availability of highly reliable predictive models for in silico ADMET assessment, we evaluated the drug-likeness profile of the selected lead compounds (CHEMBL3642843 and CHEMBL2058939) along with masitinib (Supplementary Tables 1–7). Although the predictive model highlighted a few violations for each assessed compound, including masitinib, the collective output suggests that the compounds possess an acceptable safety profile for drug-like molecules and, as such, can be recommended for further experimental assessment.

Molecular dynamics simulation, in addition to its usage for the analysis of the dynamic movement of atoms, is also an important tool in drug discovery for the analysis of protein stability changes upon ligand binding^79^. The effect of the two selected lead compounds, with masitinib as a reference, on the stability profile of the SARS-CoV-2 3CLpro was evaluated through different post-simulation analyses upon completion of a 300 ns molecular dynamics simulation protocol (Figs. 4, 5, 6, 7, 8, Supplementary Figs. 6–11). Analysis of the molecular dynamics trajectories of CHEMBL3642843 in complex with the 3CLpro suggests that the compound might show lesser activity against the viral protease, as no significant deviations to depict destabilization of the 3CLpro were observed. Although masitinib has demonstrated potency experimentally against the SARS-CoV-2 3CLpro^23^, analysis of its molecular dynamics simulation trajectory in complex with the 3CLpro also showed appreciable deviations. However, the distinct pattern of deviation observed from the trajectories of masitinibL in complex with 3CLpro strongly suggests that the lead compound might be a promising therapeutic candidate for the ravaging COVID-19.

In any attempt to repurpose, discover, or develop novel bioactive molecules, the identification of targeted macromolecules using ligand-based target prediction approaches is an essential step^80^. Although masitinib has shown desirable inhibitory effects against the SARS-CoV-2 3CLpro, its inhibitory activity against tyrosine kinases, which results in several undesirable side effects, remains a major concern. Prediction of novel targets for the selected lead compounds is therefore an important screening approach to identify the most ideal analog of masitinib. MasitinibL was the only one of the three drug-like compounds that was predicted to have a low preference for kinases in general and no predicted anti-tyrosine kinase activity (Fig. 9A–C, Supplementary Fig. 10–12). This, in addition to previous analyses, strongly suggests that masitinibL might be the most ideal masitinib analog for the inhibition of the SARS-CoV-2 3CLpro.

## Limitation

The limitation of this study is that its conclusion is still relatively preliminary. By subjecting masitinibL (the in silico-validated molecule) to in vitro and in vivo tests and, following the success of those, by evaluating the efficacy of the molecule in clinical trials in humans, the scope of this study can be expanded to overcome the limitation.

## Conclusion

The results described herein were obtained via a comprehensive in silico evaluation approach in which a potential novel drug-like candidate for the inhibition of the SARS-CoV-2 3CLpro was discovered. A set of pharmacophore-matching compounds was passed through an exhaustive virtual screening workflow, out of which masitinibL (ranked 10th) was identified. The lead compound showed a comparable interaction profile with masitinib and likewise displayed both an acceptable drug-likeness profile and promising inhibitory potentials against the viral protease. An earlier report on the efficacy of masitinib in effectively inhibiting the SARS-CoV-2 3CLpro highlighted the need for the development of analogs of masitinib with low inhibitory activity against tyrosine kinases in order to avoid the side effects that might be linked to such inhibition. Our target prediction study additionally suggested that masitinibL might be that ideal candidate, with high inhibitory activity against 3CLpro and low anti-tyrosine kinase activity. However, as this study is based on comprehensive computational evaluations, additional experimental studies are required for the validation of the suggested inhibitory activity of the lead compound.

## Supplementary Information

Supplementary Information.

## Data Availability

The datasets used and/or analysed during the current study available from the corresponding author on reasonable request.
