# Supplementary material for: MasitinibL shows promise as a drug-like analog of masitinib that elicits comparable SARS-Cov-2 3CLpro inhibition with low kinase preference
Source: Sci Rep. 2023 Apr 28;13:6972. doi: 10.1038/s41598-023-33024-2 (PMC10141821; doi:10.1038/s41598-023-33024-2)
Supplement: Supplementary file 1 — Supplementary Information. [file 41598_2023_33024_MOESM1_ESM.docx]

**Supplementary information**

**MasitinibL shows promise as a drug-like analog of masitinib that elicits comparable SARS-Cov-2 3CLpro inhibition with low kinase preference**

**
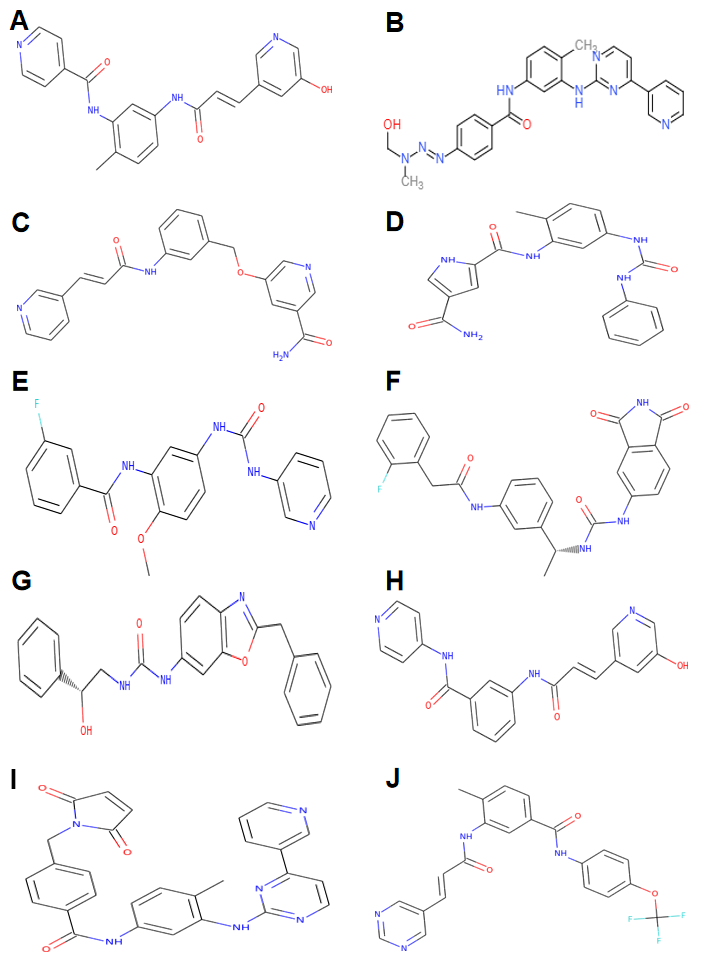
**

**Figure 1:** 2-dimensional structures of the top 10 compounds with reference to the MM-GBSA ranking. (A) MCULE-1361639875, (B) CHEMBL230286, (C) CHEMBL3805890, (D) CSC097356065, (E) MCULE-6370590876, (F) 519959077, (G) MCULE-6357305429, (H) MCULE-8253455899, (I) CHEMBL3642843, (J) CHEMBL2058939.


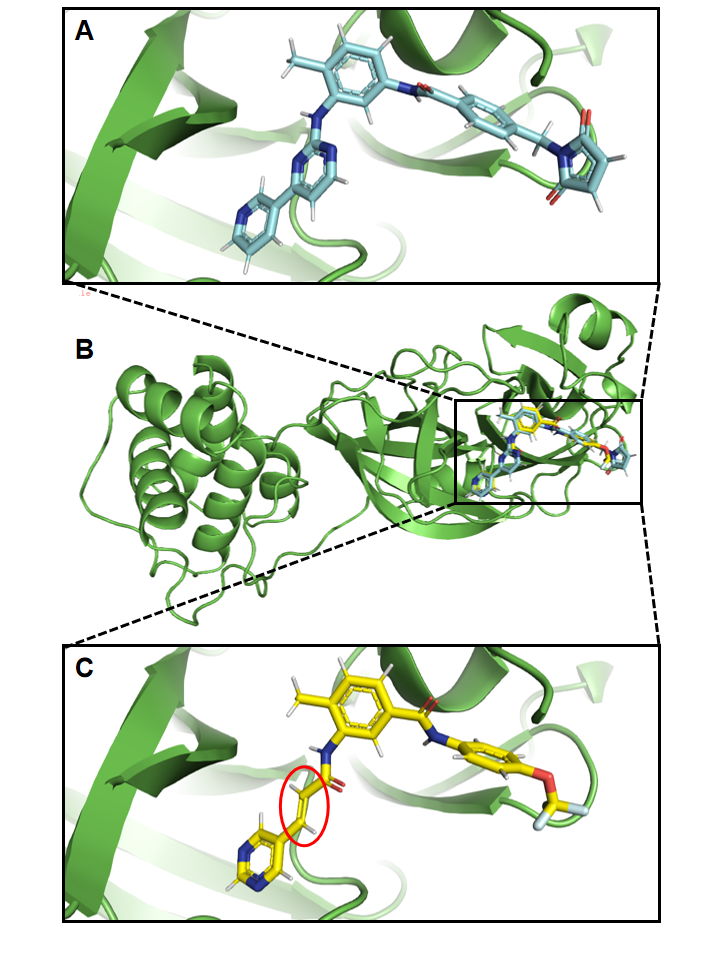


**Figure 2:** Binding pose elucidation of (A) CHEMBL3642843, and (C) CHEMBL2058939 in the catalytic pocket of the SARS-CoV-2 3CLpro. The Trans isometric geometry of CHEMBL2058939 is also highlighted in red ring. (B) shows the complete structure of the protein-ligand interactions with both ligands merged in the catalytic pocket.


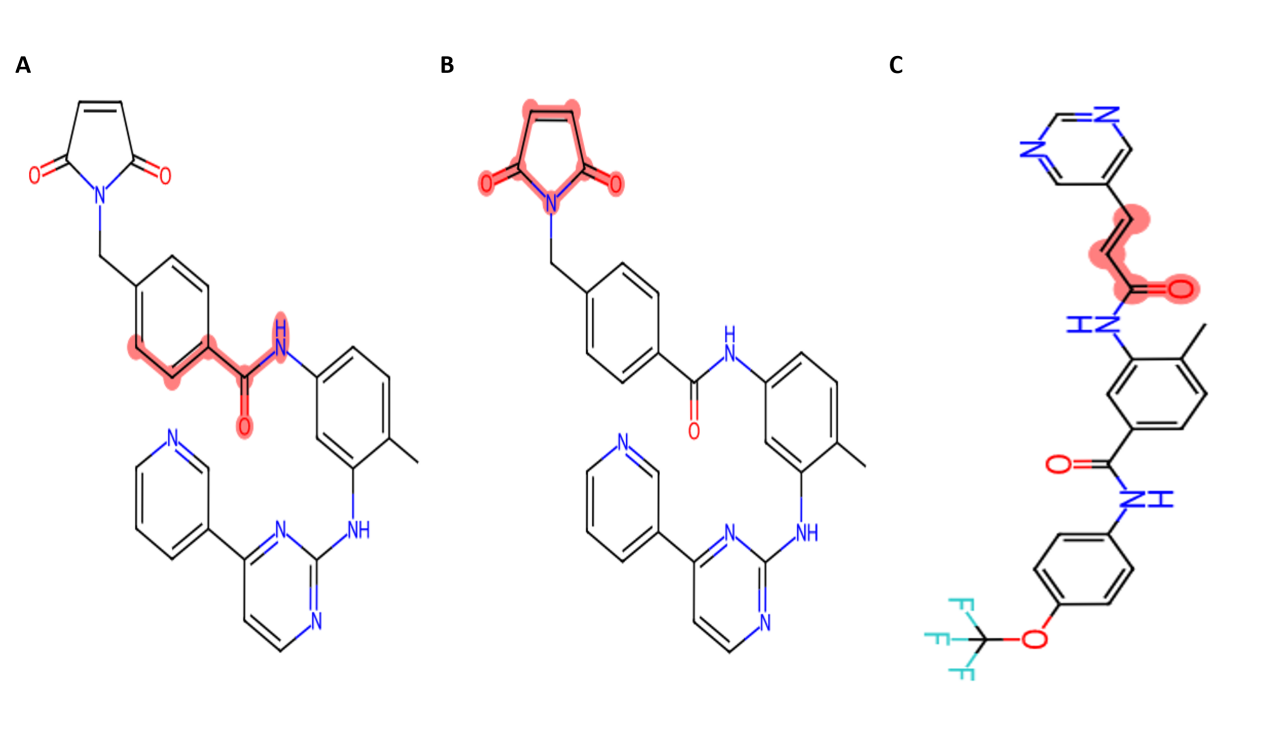


**Figure 3:** Identification of structural alerts in (A) and (B) CHEMBL3642843, and (C) CHEMBL2058939.

**
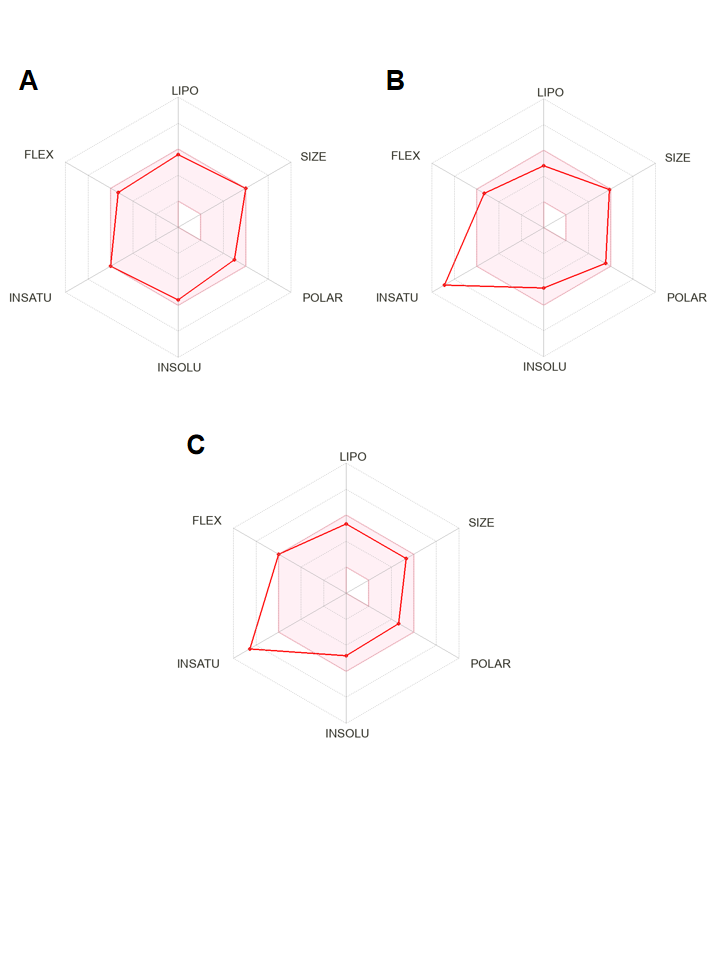
**

**Figure 4:** Bioavailability Radar displaying rapid drug-likeness appraisal for (A) masitinib, (B) CHEMBL3642843, and (C) CHEMBL2058939. Different physicochemical properties are considered, including saturation, flexibility, solubility, polarity, size, and lipophilicity. On each axis, the physiochemical range is defined by the the pink region in which the plotted radar of each compound has to fit to be considered drug-like.

**
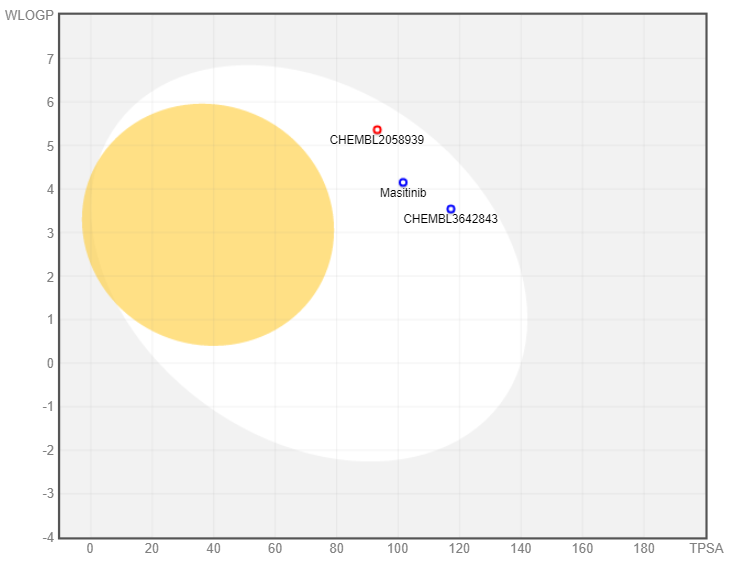
**

**Figure 5:** BOILED-Egg plot to show the blood-brain barrier permeation and passive gastrointestinal absorption propensity of masitinib, CHEMBL3642843, and CHEMBL2058939. The plot conceptually relies on two physicochemical descriptors (the apparent polarity and lipophilicity). The yolk region of the plot is the physicochemical space representing the high probability for blood-brain barrier permeation, while the egg white region is the physicochemical space representing high probability for gastrointestinal absorption. The blue and red color dots for each compound in the plot represent substrate and non-substrate of the P-glycoprotein respectively.

**
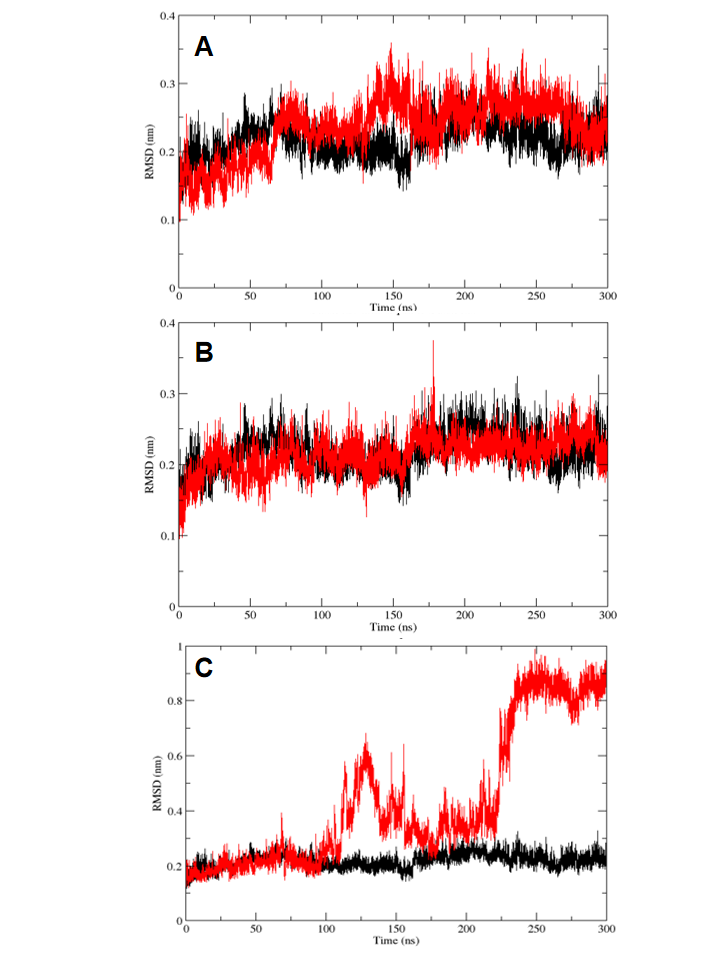
**

**Figure 6:** Individual root mean square deviation plots for the SARS-CoV-2 3CLpro in complex with (A) masitinib, (B) CHEMBL3642843, and (C) CHEMBL2058939. The trajectory of the apo protein is shown in black color in all plots.

**
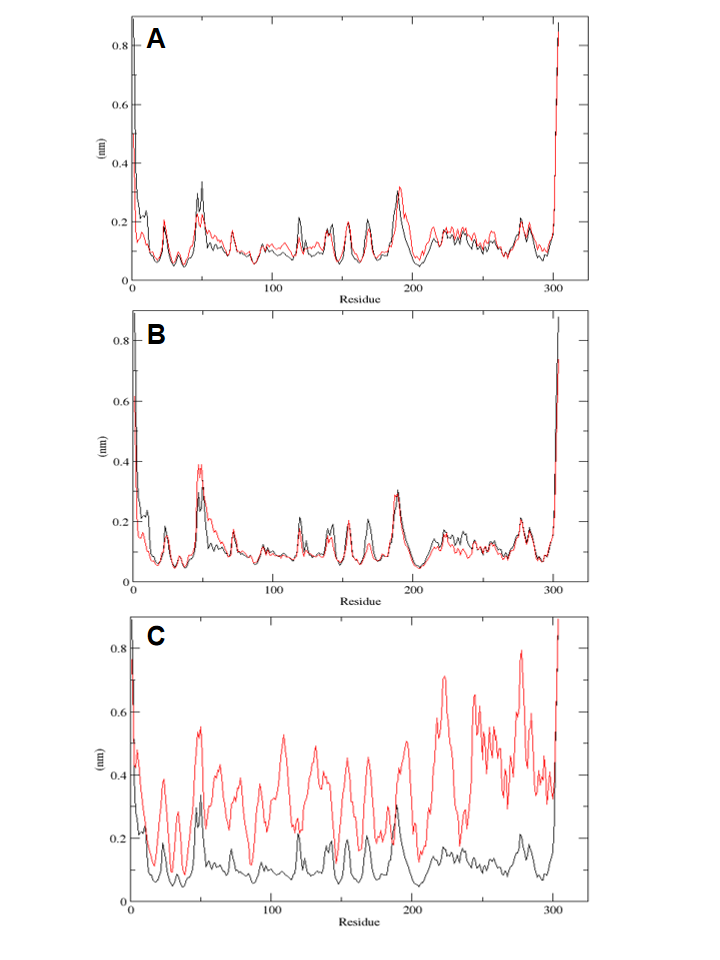
**

**Figure 7:** Individual root mean square fluctuation plots for the SARS-CoV-2 3CLpro in complex with (A) masitinib, (B) CHEMBL3642843, and (C) CHEMBL2058939. The trajectory of the apo protein is shown in black color in all plots.

**
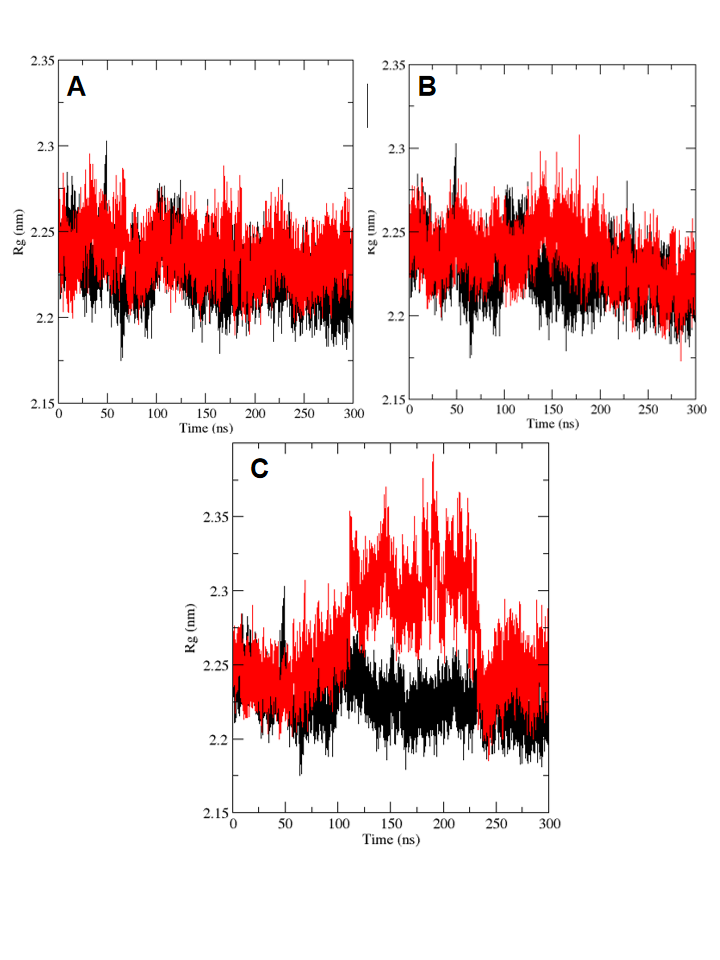
**

**Figure 8:** Individual radius of gyration plots for the SARS-CoV-2 3CLpro in complex with (A) masitinib, (B) CHEMBL3642843, and (C) CHEMBL2058939. The trajectory of the apo protein is shown in black color in all plots.

**
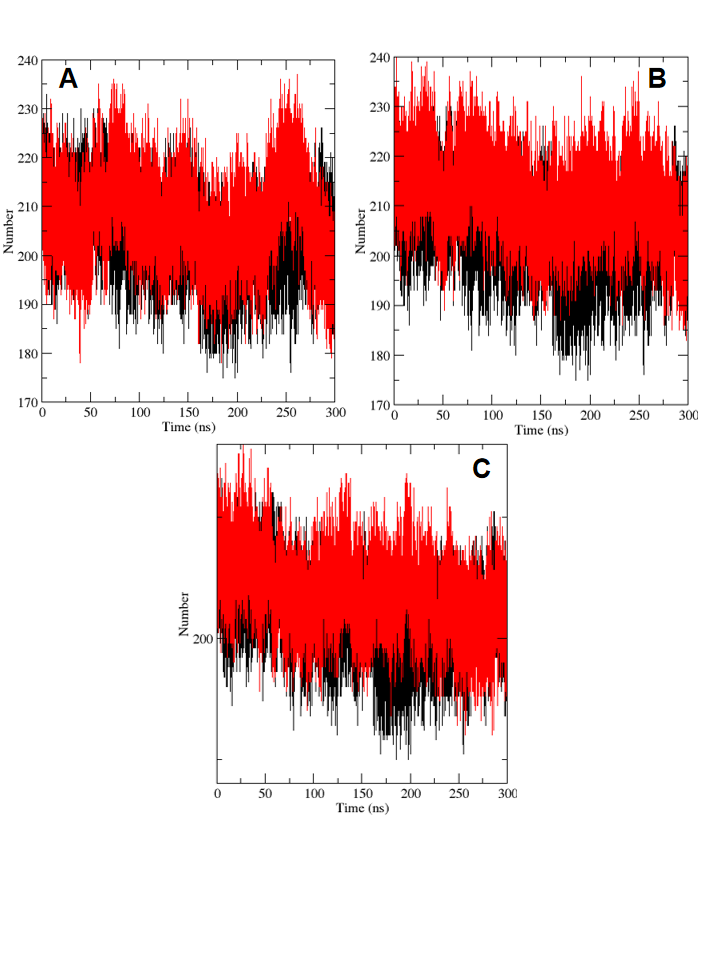
**

**Figure 9:** Individual hydrogen bond plots for the SARS-CoV-2 3CLpro in complex with (A) masitinib, (B) CHEMBL3642843, and (C) CHEMBL2058939. The trajectory of the apo protein is shown in black color in all plots.

**
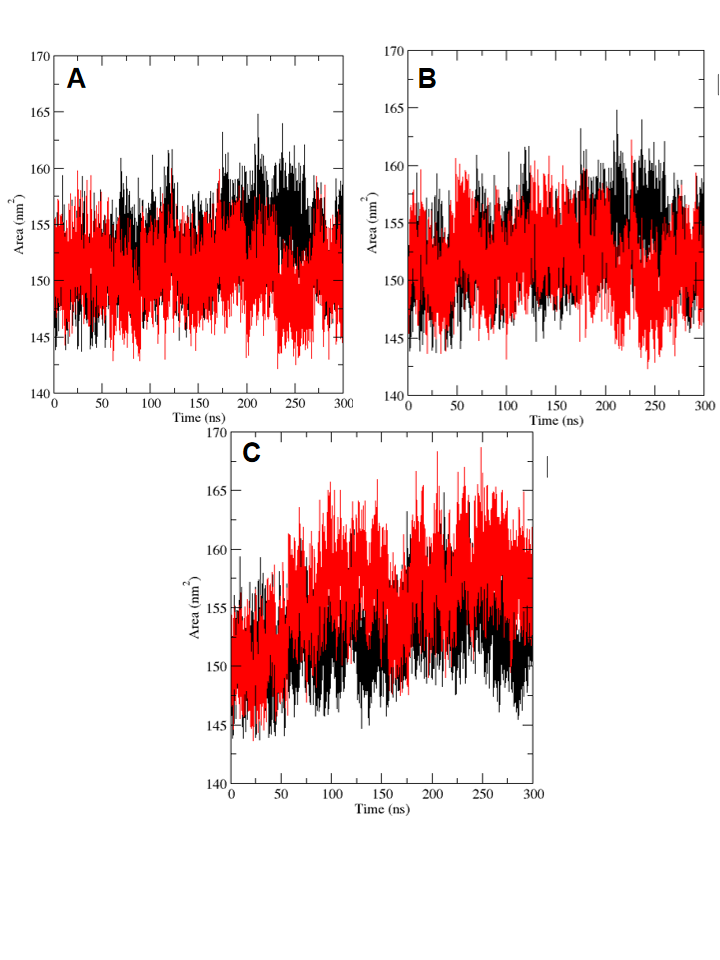
**

**Figure 10:** Individual solvent accessible surface area plots for the SARS-CoV-2 3CLpro in complex with (A) masitinib, (B) CHEMBL3642843, and (C) CHEMBL2058939. The trajectory of the apo protein is shown in black color in all plots.

**
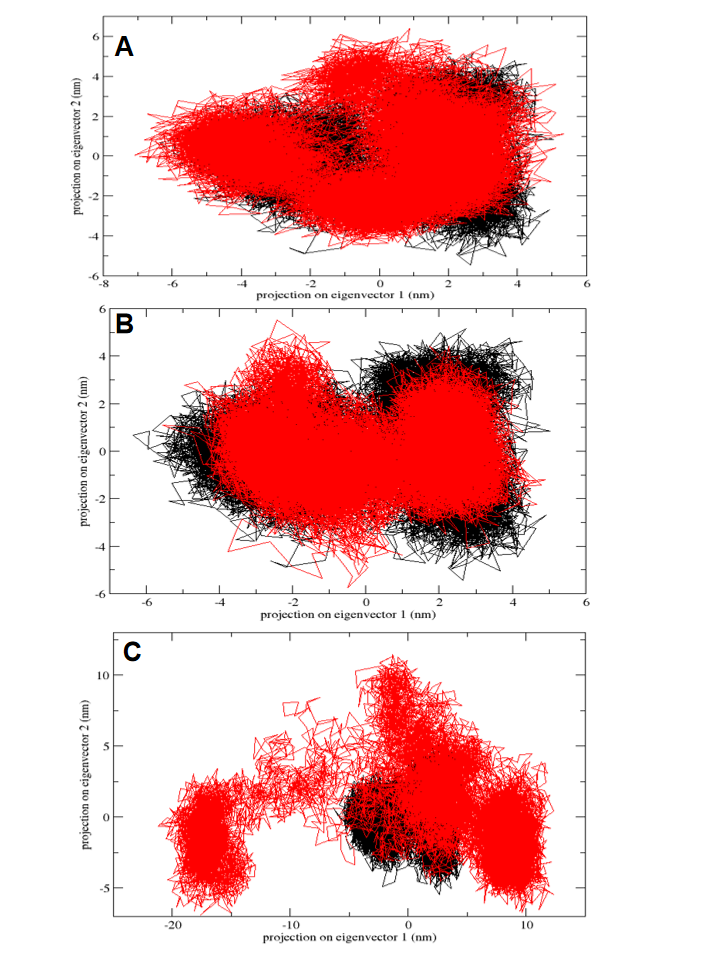
**

**Figure 11:** Individual principal component analysis plots for the SARS-CoV-2 3CLpro in complex with (A) masitinib, (B) CHEMBL3642843, and (C) CHEMBL2058939. The trajectory of the apo protein is shown in black color in all plots.

**
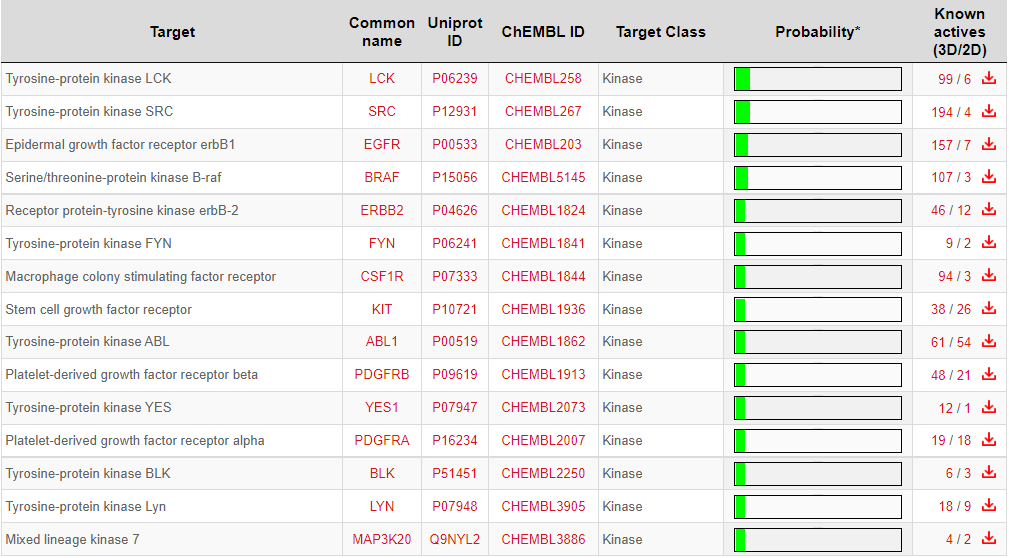
**

**Figure 12:** Top 15 biomolecular targets of masitinib as predicted by the SwissTargetPrediction tool.

**
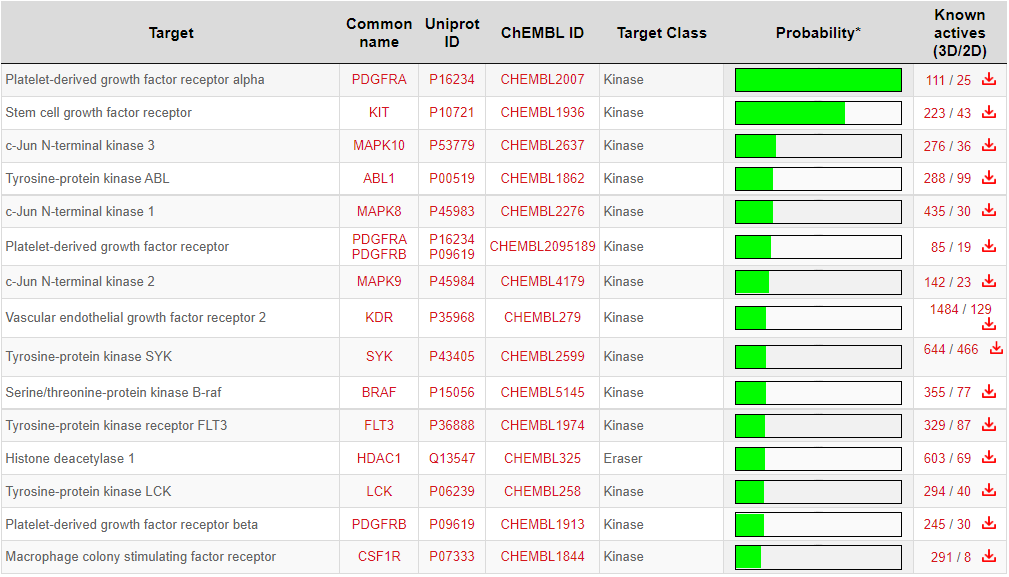
**

**Figure 13:** Top 15 biomolecular targets of CHEMBL3642843 as predicted by the SwissTargetPrediction tool.

**
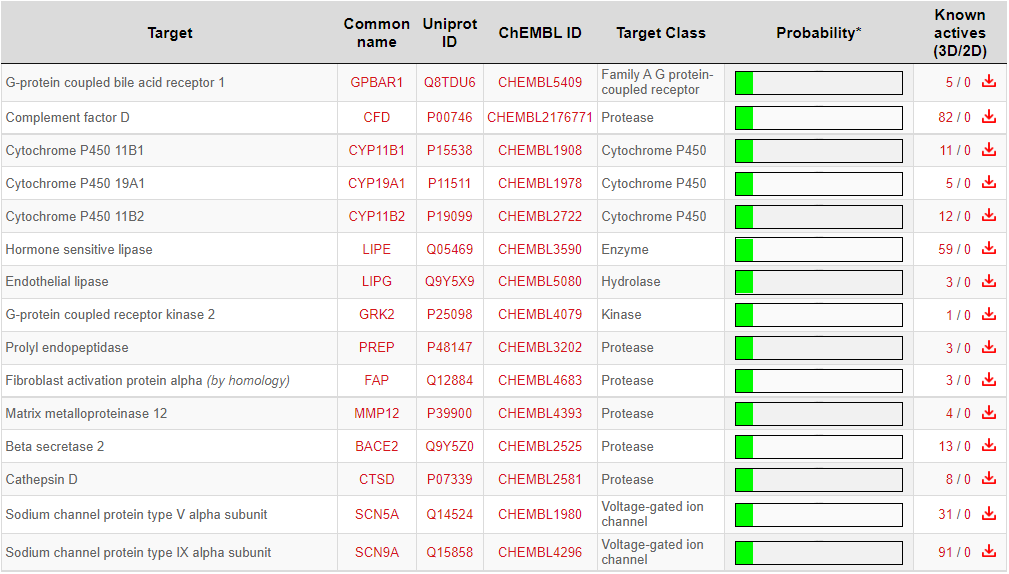
**

**Figure 14:** Top 15 biomolecular targets of CHEMBL2058939 as predicted by the SwissTargetPrediction tool.


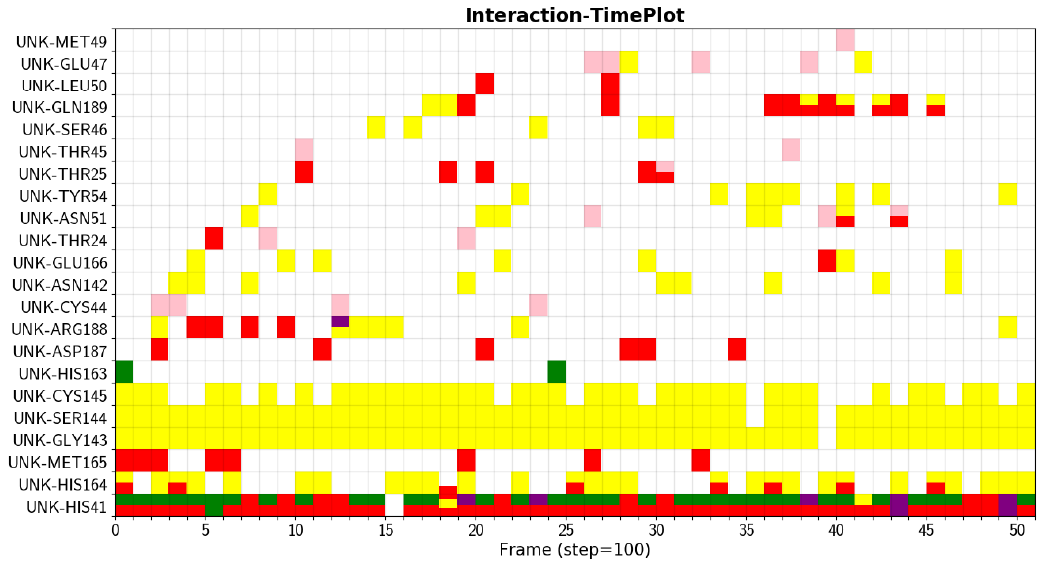


**Figure 15:** 2D interaction time plot of CHEMBL2058939 in complex with the SARS-CoV-2 3CLpro active site. The red, yellow, green, and purple colors represent the hydrophobic interactions, hydrogen bonds, pi stacking interactions, and pi cation interactions respectively.

**Table 1:** Predicted physicochemical properties of masitinib, CHEMBL3642843 and CHEMBL2058939.

|  | **Masitinib** | **CHEMBL3642843** | **CHEMBL2058939** |
| --- | --- | --- | --- |
| **Formula** | C_28_H_30_N_6_OS | C_28_H_22_N_6_O_3_ | C_22_H_17_F_3_N_4_O_3_ |
| **Molecular weight** | 498.64 g/mol | 490.51 g/mol | 442.39 g/mol |
| **Num. heavy atoms** | 36 | 37 | 32 |
| **Num. arom. heavy atoms** | 23 | 24 | 18 |
| **Fraction Csp3** | 0.25 | 0.07 | 0.09 |
| **Num. rotatable bonds** | 8 | 8 | 9 |
| **Num. H-bond acceptors** | 5 | 6 | 8 |
| **Num. H-bond donors** | 2 | 2 | 2 |
| **Molar Refractivity** | 154.58 | 142.80 | 111.82 |
| **TPSA** | 101.63 Å^2^ | 117.18 Å^2^ | 93.21 Å^2^ |

**Table 2:** Predicted lipophilicity properties of masitinib, CHEMBL3642843 and CHEMBL2058939.

|  | **Masitinib** | **CHEMBL3642843** | **CHEMBL2058939** |
| --- | --- | --- | --- |
| **Log P_O/W_ (iLOGP)** | 4.35 | 3.16 | 3.34 |
| **Log P_O/W_ (XLOGP3)** | 4.27 | 2.92 | 3.84 |
| **Log P_O/W_ (WLOGP)** | 4.15 | 3.54 | 5.36 |
| **Log P_O/W_ (MLOGP)** | 2.37 | 1.78 | 1.90 |
| **Log P_O/W_ (SILICOS-IT)** | 4.89 | 3.45 | 3.95 |
| **Consensus Log P_O/W_** | 4.01 | 2.97 | 3.68 |

**Table 3:** Predicted water solubility properties of masitinib, CHEMBL3642843 and CHEMBL2058939.

|  | **Masitinib** | **CHEMBL3642843** | **CHEMBL2058939** |
| --- | --- | --- | --- |
| **Log S (ESOL)**  **Solubility**  **Class** | -5.57  1.35e-03 mg/ml  Moderately soluble | -4.67  1.04e-02 mg/ml  Moderately soluble | -4.82  6.63e-03 mg/ml  Moderately soluble |
| **Log S (Ali)**  **Solubility**  **Class** | -6.12  3.81e-04 mg/ml  Poorly soluble | -5.04  4.45e-03 mg/ml  Moderately soluble | -5.49  1.42e-03 mg/ml  Moderately soluble |
| **Log S (SILICOS-IT)**  **Solubility**  **Class** | -9.31  2.45e-07 mg/ml  Poorly soluble | -9.26  2.68e-07 mg/ml  Poorly soluble | -7.67  9.37e-06 mg/ml  Poorly soluble |

**Table 4:** Predicted pharmacokinetic properties of masitinib, CHEMBL3642843 and CHEMBL2058939.

|  | **Masitinib** | **CHEMBL3642843** | **CHEMBL2058939** |
| --- | --- | --- | --- |
| **GI absorption** | High | High | High |
| **BBB permeant** | No | No | No |
| **P-gp substrate** | Yes | Yes | No |
| **CYP1A2 inhibitor** | No | No | Yes |
| **CYP2C19 inhibitor** | Yes | Yes | Yes |
| **CYP2C9 inhibitor** | Yes | Yes | Yes |
| **CYP2D6 inhibitor** | Yes | No | Yes |
| **CYP3A4 inhibitor** | Yes | Yes | Yes |
| **Log K_P_ (skin permeation)** | -6.31 cm/s | -7.22 cm/s | -6.27 cm/s |

**Table 5:** Predicted drug-likeness properties of masitinib, CHEMBL3642843 and CHEMBL2058939.

|  | **Masitinib** | **CHEMBL3642843** | **CHEMBL2058939** |
| --- | --- | --- | --- |
| **Lipinski** | Yes: 0 violation | Yes: 0 violation | Yes: 0 violation |
| **Ghose** | No; 2 violations | No; 2 violations | Yes: 0 violation |
| **Veber** | Yes: 0 violation | Yes: 0 violation | Yes: 0 violation |
| **Egan** | Yes: 0 violation | Yes: 0 violation | Yes: 0 violation |
| **Muegge** | Yes: 0 violation | Yes: 0 violation | Yes: 0 violation |
| **Bioavailability score** | 0.55 | 0.55 | 0.55 |

**Table 6:** Predicted medicinal chemistry properties of masitinib, CHEMBL3642843 and CHEMBL2058939.

|  | **Masitinib** | **CHEMBL3642843** | **CHEMBL2058939** |
| --- | --- | --- | --- |
| **PAINS** | 0 alert | 0 alert | 0 alert |
| **Brenk** | 0 alert | 1 alert | 1 alert |
| **Leadlikeness** | No; 3 violations | No; 2 violations | No; 3 violations |
| **Synthetic accessibility** | 3.99 | 3.60 | 2.90 |

**Table 7:** Oral toxicity and toxicity endpoint prediction for masitinib, CHEMBL3642843 and CHEMBL2058939.

|  | **Masitinib** | **CHEMBL3642843** | **CHEMBL2058939** |
| --- | --- | --- | --- |
| **LD50** | 1000 mg/kg | 840 mg/kg | 800 mg/kg |
| **Toxicity class** | 4 | 4 | 4 |
| **Hepatotoxicity/Probability** | Active/0.55 | Inactive/0.55 | Active/0.64 |
| **Carcinogenicity/Probability** | Inactive/0.63 | Active/0.57 | Active/0.54 |
| **Immunotoxicity/Probability** | Inactive/0.95 | Active/0.77 | Inactive/0.93 |
| **Mutagenicity/Probability** | Inactive/0.68 | Inactive/0.70 | Inactive/0.72 |
| **Cytotoxicity/Probability** | Inactive/0.60 | Inactive/0.73 | Inactive/0.70 |
